# Overt and Occult Hepatitis B in Hemodialysis Patients: Insights from High-Sensitivity Testing and Genetic Analysis

**DOI:** 10.3390/diagnostics16213588

**Published:** 2026-10-30

**Authors:** Yulia V. Ostankova, Elena N. Serikova, Alexander N. Shchemelev, Marina B. Kusevitskaya, Maksim V. Sannikov, Olga A. Gorskaya, Valentina V. Basina, Leonid Y. Kusevitskiy, Ilya A. Mashkov, Elena D. Pshenay-Severin, Nadezhda A. Pechnikova, Edward S. Ramsay, Areg A. Totolian

**Affiliations:** 1 Saint Petersburg Pasteur Institute, 14 Mira Street, 197101 St. Petersburg, Russia; genista.bio@gmail.com (E.N.S.); tvildorm@gmail.com (A.N.S.); nikanobelevka@gmail.com (N.A.P.); totolian@spbraaci.ru (A.A.T.); 2 St. Petersburg State Institution of Health “City Clinical Hospital No 31”, 197110 St. Petersburg, Russia; mbkus@mail.ru; 3 Nikiforov Russian Center of Emergency and Radiation Medicine, EMERCOM of Russia, 4/2 Academica Lebedeva Street, 194044 St. Petersburg, Russia; smakv@mail.ru; 4 D.O. Ott Research Institute of Obstetrics, Gynaecology and Reproduction, 3 Mendeleevskaya Line, 199034 St. Petersburg, Russia; gorskaya70@rambler.ru; 5 Department of Adult Infectious Diseases and Epidemiology, Saint Petersburg State Pediatric Medical University, 2 Litovskaya Street, 194100 St. Petersburg, Russia; v.basina@mail.ru (V.V.B.); e.pshenay@mail.ru (E.D.P.-S.); 6 Federal State Budgetary Educational Institution of Higher Education “Academician I.P. Pavlov First St. Petersburg State Medical University” of the Ministry of Healthcare of Russian Federation, 197022 St. Petersburg, Russia; stomdoc@mail.ru; 7 St. Petersburg State Budgetary Institution of Health “City Polyclinic No 99”, 38 Yesenina Street, Bldg. 1, 194358 St. Petersburg, Russia; ilya.mashkov@list.ru

**Keywords:** hepatitis B virus (HBV), occult hepatitis B infection (OBI), hemodialysis, nested real-time PCR, molecular diagnostics, genotyping, S-gene mutations

## Abstract

**Background/Objectives:** Occult hepatitis B infection (OBI) remains a diagnostic challenge, particularly in hemodialysis (HD) patients, who are vulnerable to HBV infection due to immune dysfunction and repeated exposure. **Methods:** We evaluated the prevalence and molecular characteristics of OBI among 874 HD patients in St. Petersburg, Russia, using high-sensitivity nested real-time PCR targeting the S and X genes. **Results:** HBV DNA was detected in 3.7% (32/874), as reflected in subcategories: overt CHB in 2.1% (18/874) and OBI in 1.6% (14/874). Genotype D predominated (93.8%), with subgenotypes D2 (56.3%), D1 (25.0%), and D3 (12.5%) present. OBI patients had low viral loads (6.0–148.3 IU/mL), whereas CHB patients showed high viremia (7843–64,870,000 IU/mL). Immune escape mutations were significantly more frequent in OBI (92.9%) than CHB (16.7%; *p* < 0.0001), with A128V being most common (31.3%). Core mutations were nearly universal (93.7%). Drug resistance mutations were found in 6.3% of cases without prior antiviral therapy. Seronegative infections (i.e., negative for anti-HBc IgG and anti-HBs IgG) were common, comprising 77.8% of CHB and 14.3% of OBI cases. Anti-HBc prevalence increased with dialysis vintage (3.8% to 43.1%, *p* < 0.0001), while anti-HBs declined with age (rs = −0.85, *p* = 0.0001). **Conclusions:** In conclusion, OBI affects 1.6% of HD patients in this cohort, with immune escape mutations as a key molecular driver of the occult phenotype. These findings suggest that HBV DNA screening may warrant consideration in HD patients, as serological results alone are inadequate for ruling out occult infection.

## 1. Introduction

Hepatitis B virus (HBV) infection remains one of the leading causes of chronic liver disease, cirrhosis, and hepatocellular carcinoma worldwide [1].

A particularly challenging, and often overlooked, form of infection is occult hepatitis B infection (OBI). It is defined as: the presence of replication-competent HBV DNA in the liver and/or serum in the absence of detectable hepatitis B surface antigen (HBsAg) by standard serological assays, with or without antibodies against HBV core or surface antigens (anti-HBc and/or anti-HBs) [2,3]. Approximately 20% of OBI cases are seronegative for all conventional HBV markers, rendering them invisible to routine screening [3,4]. OBI is not a benign condition. It can contribute to the progression of liver fibrosis, maintain the pro-oncogenic properties of HBV, and lead to hepatocellular carcinoma, even in the absence of detectable HBsAg [5,6,7]. Importantly, OBI can reactivate under immunosuppressive conditions, with clinical manifestations ranging from asymptomatic elevations of aminotransferases to acute hepatitis, liver failure, and fulminant hepatitis. This risk applies to a broad range of immunocompromised individuals, including hemodialysis patients, recipients of solid organ or hematopoietic stem cell transplants, patients receiving immunosuppressive or anticancer therapy, and those with HIV infection [7,8]. Furthermore, OBI carries a potential risk of viral transmission through blood transfusion, dialysis procedures, and organ transplantation [4].

Patients on maintenance hemodialysis (HD) represent a uniquely vulnerable population for both overt and occult HBV infection. Several converging factors contribute to this heightened risk: repeated vascular access, shared dialysis equipment, frequent blood transfusions, imperfect infection control practices, and uremia-associated immune dysfunction [4,9,10]. Once infected, HD patients are significantly more likely to become chronic HBV carriers (up to 60% vs. 5–10% in the general population) due to impaired immune responses, and this chronic infection is associated with increased risks of hepatocellular carcinoma and overall mortality [9,11,12,13,14,15]. The prevalence of OBI in HD patients varies markedly between geographical regions, ranging from 0% to 58% in published reports, depending on HBV endemicity, sample size, and the sensitivity of the molecular methods used [16,17,18,19,20,21,22]. Therefore, accurate identification of OBI in this population is not merely an epidemiological exercise, but a critical component of clinical management and transplantation safety [7,23,24].

The mechanisms underlying OBI are heterogeneous. In some cases, OBI results from epigenetic silencing of cccDNA or host immune-mediated suppression of viral gene expression, leading to low or undetectable HBsAg production [4]. In other cases, mutations in the S gene impair HBsAg detection or secretion. Depending on the mechanism and the level of viral replication, OBI can be classified as true OBI, characterized by low or undetectable viral load (typically below 200 IU/mL), or as «false» OBI, in which HBsAg is undetectable due to mutations but viral replication exceeds 200 IU/mL, resembling overt infection. Importantly, mutations in the S gene are not exclusive to «false» OBI; they are frequently found in true OBI as well. Thus, S gene mutations represent one of several mechanisms that can lead to the OBI phenotype. Both forms pose diagnostic challenges. Viral loads in true OBI frequently fall below the detection limit of most commercial PCR assays [2,7,25]. In addition to nucleic acid testing, ultrasensitive quantitative HBsAg assays with a lower limit of detection as low as 0.005 IU/mL have been developed and can identify OBI cases missed by conventional assays [26,27,28]. However, their sensitivity remains insufficient to replace HBV DNA testing in all cases. Furthermore, S gene mutations—particularly within the major hydrophilic region (MHR) that contains the immunodominant «a» determinant—can impair HBsAg detection by immunoassays and reduce amplification efficiency in single-target PCR methods [29,30,31]. In HD patients with OBI, specific mutations in the S protein within the «a» determinant of the major hydrophilic loop (MHL, SHBs aa 100–165) have been identified, including T113A/S, T118K/M, P120Q/S/T, T123I, C124F, P127T, Q129R, T131N, M133S, F134L, D144E, P153L, Y161F/S; and within the internal loop of the SHBs protein (aa 27–79), including N40S, G44E, Q51R/P; and in the C-terminal region (aa 170–226), including F170S [17,32,33,34,35,36,37]. Mutations in the transmembrane domain of HBsAg have also been shown to contribute to OBI development [33]. Even the classical immune escape mutation G145R, which renders HBsAg undetectable by most serological assays, has been documented in HD patients [38,39]. Furthermore, mutations in the polymerase, core, and X regions may co-occur and further complicate molecular detection [40].

To overcome these diagnostic limitations, the international consensus (Taormina expert meeting, 2019) recommends the use of highly sensitive nucleic acid amplification techniques, ideally nested PCR targeting at least two independent genomic regions, for reliable OBI detection [3]. Various nested PCR protocols have been successfully applied for OBI screening in high-risk populations, including HD patients [19,22,41,42,43,44,45]. The use of two viral gene targets significantly reduces the risk of false-negative results due to mutations in a single primer binding site. Moreover, when OBI isolates are detected, subsequent genotyping and mutational analysis provide essential insights into the molecular epidemiology of HBV in dialysis centers, inform vaccination strategies, and help predict the risk of immune escape and antiviral resistance.

From an infection control perspective, the distinction between overt and occult HBV infection in hemodialysis units is critically important. HBsAg-positive patients (overt infection) are routinely identified through standard serological screening and are typically dialyzed in dedicated isolation rooms or on dedicated machines to prevent viral transmission. Therefore, comprehensive molecular screening (targeting both overt and occult infections) is essential not only for individual patient management, but also for the safety of the entire dialysis unit.

This study aimed to evaluate the diagnostic value of a highly-sensitive, nested real-time PCR assay for detection of hepatitis B virus DNA, while gaining valuable data for insight into several areas. These include: identifying OBI in patients undergoing hemodialysis therapy; and determining the genetic characteristics (genotypes, mutations) of the identified isolates representing different manifestations (overt, occult).

## 2. Materials and Methods

### 2.1. Study Design and Ethical Approval

The study material consisted of plasma samples obtained from selected patients on maintenance hemodialysis residing in St. Petersburg, Russia, collected during routine medical examinations at healthcare facilities. The study protocol was approved by the Local Ethics Committee of the St. Petersburg Pasteur Institute (Protocol No. 67, dated 22 February 2017, Protocol No. 97, dated 29 January 2020, and Protocol No. 88, dated 3 October 2023). All participants were informed about the purpose and methodology of the study and provided written informed consent.

### 2.2. Study Population

A total of 874 patients on maintenance hemodialysis were enrolled in the study. Inclusion criteria were as follows: age ≥18 years, receipt of maintenance hemodialysis for at least 3 months, vaccination against HBV prior to (or at the initiation of) hemodialysis therapy, availability of complete medical records (including age, sex, duration of hemodialysis therapy, and the underlying disease leading to hemodialysis initiation), written informed consent to participate in the study, and written informed consent to provide a venous blood sample. Exclusion criteria were as follows: documented HIV infection; acute hepatitis B or C within the previous 6 months; hepatitis B vaccination within 30 days prior to enrollment; history of kidney or liver transplantation; transfusion of red blood cells, fresh frozen plasma, or platelet concentrate within 3 months prior to enrollment; receipt of anti-HBV immunoglobulin or nucleos(t)ide analogues (entecavir, tenofovir) within 6 months; refusal to participate; or inability to obtain a blood sample.

**Figure 1:**
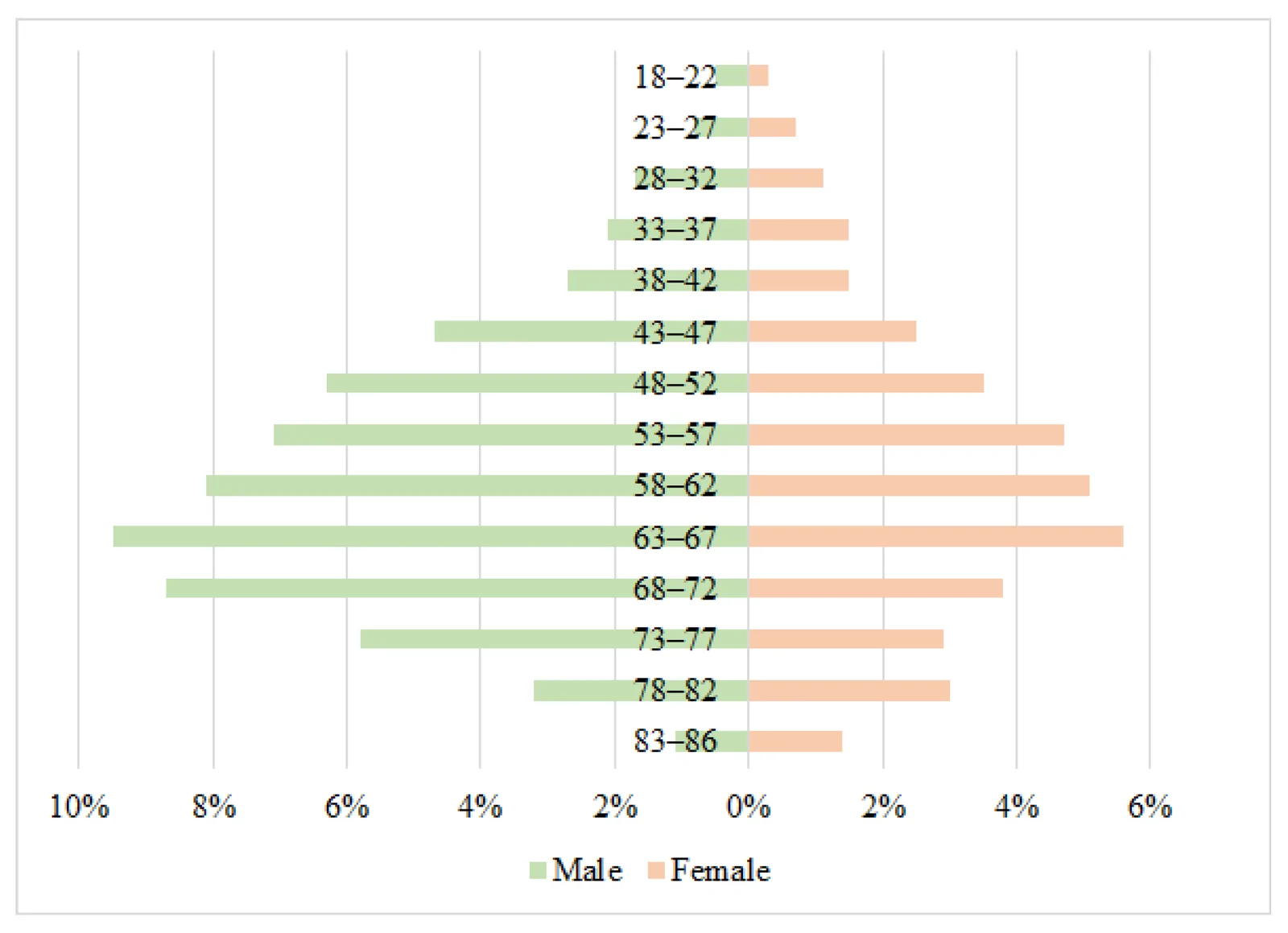
Age and sex distribution of the study population (*N* = 874).

The overall study population consisted of 545 men (62.4%) and 329 women (37.6%). Participant ages ranged from 18 to 86 years, with a mean of 59.06 years. Among male patients, age ranged from 18 to 83 years (mean 59.04 years). Among female patients, age ranged from 18 to 86 years (mean 59.10 years). The detailed age and sex distribution of the cohort is presented in Figure 1.

The underlying causes of end-stage renal disease (ESRD) were categorized into four groups. Diabetes mellitus (diabetic nephropathy), the most common underlying cause accounting for 343 patients (39.2%), was designated as group 1. Arterial hypertension (hypertensive nephrosclerosis), accounting for 219 patients (25.1%), was designated as group 2. Primary kidney disease (glomerulonephritis, polycystic kidney disease, other intrinsic renal disorders), accounting for 194 patients (22.2%), was designated as group 3. Other causes (vasculitis, hereditary diseases, malignancies, drug-induced nephropathies), covering the remaining 118 patients (13.5%), were designated as group 4.

The duration of hemodialysis therapy was divided into four categories: <1 year (*n* = 78, 8.9%), 1 to 5 years (*n* = 378, 43.2%), >5 to 10 years (*n* = 360, 41.2%), and >10 years (*n* = 58, 6.6%). These demographic and clinical characteristics formed the basis for subsequent serological and molecular analyses.

### 2.3. Sample Transportation and Storage

For serological and molecular biological testing of viral hepatitis markers, we used blood plasma collected from the antecubital vein into sterile disposable K_2_-EDTA anticoagulant tubes after overnight fasting. Plasma separation was achieved by centrifugation at 4 °C (1000× *g*) for 10 min. To prevent false-negative results in low viral load cases, all samples underwent preliminary viral concentration through ultracentrifugation (24,000× *g*, 4 °C, 1 h). Aliquots were distributed into cryovials for storage and subsequent testing: ELISA (2 × 500 μL); PCR (2 × 250 μL, 1 × 500 μL); and PCR with sequencing (5000 μL). Samples were transported within 24 h of collection using specialized biomaterial containers maintained at 4 to 8 °C.

### 2.4. Enzyme-Linked Immunosorbent Assay

Enzyme-linked immunosorbent assay (ELISA) analysis for HBV markers involved qualitative determination of HBsAg, anti-HBs IgG, and anti-HBc IgG as previously described [42,43]. Tests were repeated twice using reagents in accordance with manufacturer instructions. The following commercial kits were used: “DS-EIA-HBsAg”, “DS-EIA-ANTI-HBsAg”, and “DS-EIA-ANTI-HBc”, which detects total anti-HBc (IgM + IgG) (Diagnostic Systems RPC, Nizhny Novgorod, Russia) and “Vectohep B-HBs-antigen”, “VectoHBsAg-antibodies”, and “HepaBest anti-HBc-IgG”, which detects anti-HBc IgG specifically (Vector-Best, Novosibirsk, Russia). Initially reactive or inconclusive results for HBsAg were subjected to confirmatory testing. The “ELISA-HBsAg-confirmatory test” (Diagnostic Systems RPC, Nizhny Novgorod, Russia) was used for HBsAg neutralization. The analytical sensitivity of the HBsAg assays is 0.01 IU/mL, according to the manufacturers’ instructions. Anti-HBc results were interpreted by comparing the two assays: concordant positive results from both kits were considered indicative of anti-HBc IgG; a positive result from the total anti-HBc assay with a negative IgG result would have indicated possible IgM and required testing with a separate IgM-specific assay; a positive IgG result with a negative total anti-HBc result would have been considered inconclusive. In all tested samples, the results from both assays were concordant. Patients negative for two markers (anti-HBc IgG/IgM and anti-HBs IgG) were considered seronegative [3].

### 2.5. Nucleic Acid Extraction

DNA extraction was performed using the “NK-Magno-UltraPure-A” reagent kit (NPF Epitop, LLC, Saint Petersburg, Russia) according to manufacturer instructions, with modifications to increase the input volume when required. For initial PCR screening, DNA was extracted from 200 µL of plasma. For all samples with detectable HBV DNA, the result was confirmed by a second, independent extraction performed blinded to the first result from a reserved, previously unprocessed 200 µL aliquot of the same plasma sample. In cases where the initial PCR result was inconclusive (i.e., detection in only one of the two viral channels), DNA extraction was repeated from a larger plasma volume (500 µL) to increase the likelihood of detecting low-level viremia. For samples requiring approximate viral load quantification and subsequent sequencing, DNA was extracted from ≥5 mL of plasma. Each extraction batch included a negative control (sterile PBS).

### 2.6. HBV DNA Amplification

Our multi-step molecular detection strategy was designed to maximize the identification of low-level viremia in accordance with the recommendations of the Taormina Workshop on Occult HBV Infection. Initial screening was performed using the “AmpliSens HBV-FL real-time PCR” commercial kit (CRIE, Moscow, Russia), which has an analytical sensitivity of 100 IU/mL.

To ensure the detection of all low-viremic cases, samples were subsequently analyzed with a highly-sensitive in-house method specifically optimized for HBsAg-negative samples (5 IU/mL lower limit of detection from 200 µL of plasma) [46]. It consists of a first-round PCR with outer primers HBV longF (5′-TTTTTCACCTCTGCCTAATCA-3′) and HBV longR (5′-AAAAAGTTGCATGRTGMTGG-3′) that flank nearly the full HBV genome. This is followed by a second-round multiplex real-time PCR with two sets of inner primers and TaqMan probes targeting the S gene (FAM channel) and X gene (ROX channel) in a single tube, plus a third set targeting the human hypoxanthine-guanine phosphoribosyltransferase (HPRT) gene (internal extraction/amplification control, Cy5 channel) (Table 1).

The first round of nested PCR was performed in a total reaction volume of 25 µL. The amplification mix contained 12.5 µL of M-428 2.5× real-time PCR reaction mix (Syntol, Moscow, Russia). According to the manufacturer, this 2.5× master mix consists of a thermostable SynTaq DNA polymerase with activity-inhibiting (hot start) components, an optimized PCR buffer (containing Tris-HCl, KCl, and (NH_4_)_2_SO_4_), 2.5 mM MgCl_2_, and a 0.5 mM mixture of dNTPs. The reaction was supplemented with 1.25 µL of forward outer primer and 1.25 µL of reverse outer primer, resulting in a final concentration of 0.5 µM per primer. The thermal cycling conditions for the first round are provided in Supplementary Table S1. To prevent cross-contamination between the two amplification rounds, each first-round reaction mixture was overlaid with 25 µL of sterile mineral oil (“PCR-grade mineral oil”, Helicon, Moscow, Russia) prior to thermal cycling.

For the second round, 10 µL of the first-round amplification product was carefully aspirated from beneath the oil layer and used as template in a total reaction volume of 25 µL. The multiplex real-time PCR mix contained the same M-428 2.5× reaction mix (Syntol, Russia) with the buffer composition described above. The reaction was supplemented with 2.5 µL of a primer–probe mix containing three primer pairs and three corresponding hydrolysis probes, resulting in final concentrations of 0.4 µM per primer and 0.2 µM per probe (primer/probe sequences in Table 1). The thermal cycling conditions for the second round are provided in Supplementary Table S2. Two negative controls were included at each PCR stage. Both controls underwent the entire procedure: the second-round PCR was performed using the first-round amplification product of the negative control as template. This allowed monitoring for contamination at both the first and second amplification rounds.

Results were interpreted based on the presence or absence of a fluorescence curve crossing the threshold, set as the midpoint of the linear phase of the positive control. A sample was considered positive if Ct values were recorded in both viral channels (FAM for S gene, ROX for X gene), with the internal control (Cy5) amplifying within the expected range (Ct < 38); negative if no Ct was recorded in either viral channel, with valid internal control; inconclusive if Ct was detected in only one viral channel, in which case DNA extraction was repeated from a larger plasma volume (500 μL) as described in Section 2.5; and invalid if the internal control failed to amplify (no Ct or Ct ≥ 38), regardless of viral signals, in which case the sample was retested from the original extract or, if the control repeatedly failed, from a new plasma extraction. A case was defined as OBI when HBV DNA was detected in at least two independent PCR assays from two independently extracted aliquots of the same plasma sample, in the absence of HBsAg.

For all samples confirmed positive for HBV DNA, a semi-quantitative estimation of viral load was performed using a commercial real-time PCR kit (“AmpliSens HBV-Monitor-FL real-time PCR”, CRIE, Moscow, Russia). According to the manufacturer’s protocol, it has a stated analytical sensitivity of 15 IU/mL when DNA is extracted from 1 mL of plasma. In the present study, to increase the input volume and thus improve the likelihood of detecting low-level HBV DNA, DNA was extracted from 5 mL of plasma as described in Section 2.5. However, a formal validation of this modified protocol (extraction from 5 mL) was not performed. Therefore, the obtained values are reported as semi-quantitative estimates and should not be interpreted as absolute quantitative concentrations. For samples with viral loads below the lower limit of quantification (LLOQ) of the commercial assay (15 IU/mL), the measured concentration is reported with the prefix «<LLOQ» to indicate that the value is below the validated range of the assay. These estimates were used primarily to compare the relative viral replication levels between OBI and CHB patients and to guide subsequent sequencing. The same DNA extract was used for HBV DNA sequencing.

### 2.7. HBV Genomic Amplification and Sequencing

For sequencing, overlapping fragments covering the entire HBV genome were amplified using previously published primer pairs [47,48,49,50,51]. PCR products were purified by ethanol precipitation to remove primers, nucleotides, and buffer components. To 20 µL of PCR product, 2 µL of 3 M sodium acetate (pH 5.2) and 1 µL of glycogen (20 mg/mL) were added, followed by the addition of 60 µL of chilled 96% ethanol. The mixture was incubated at −20 °C for 30 min and then centrifuged at 16,000× *g* for 15 min at 4 °C. Supernatant was carefully discarded, and the pellet was washed with 200 µL of chilled 70% ethanol, followed by centrifugation at 16,000× *g* for 5 min at 4 °C. The supernatant was removed, and the pellet was air-dried for 5–10 min. Purified DNA was dissolved in 30 µL of TE buffer (10 mM Tris-HCl, 1 mM EDTA, pH 8.0).

Nucleic acid concentration was measured using a Qubit 2.0 fluorometer (Thermo Fisher Scientific, Waltham, MA, USA) according to the manufacturer’s standard protocol. To assess the purity and integrity of amplified products, 5 µL of purified DNA was visualized on a 1.5% agarose gel stained with ethidium bromide.

For Sanger sequencing, purified HBV DNA fragments were used at a concentration of 50–100 ng per reaction, depending on the nucleotide composition of the target region. The sequencing reaction was performed using the ABI PRISM BigDye Terminator v3.1 Cycle Sequencing Kit (Applied Biosystems, Foster City, CA, USA) according to manufacturer instructions. Both forward and reverse specific primers were used at a concentration of 1.6 pmol/µL, in triplicate for each primer pair and each analyzed region. The sequencing reaction mixture (20 µL total volume) contained: 0.2–8.0 µL of template DNA (adjusted to provide 50–100 ng), 3.0 µL of sequencing primer (1.6 pmol/µL), 8 µL of BigDye Terminator v3.1 ready reaction mix, and deionized water to volume. Thermal cycling conditions for the sequencing reaction were: 25 cycles of 96 °C for 10 s, 50 °C for 5 s, and 60 °C for 4 min.

Sequencing reaction products were purified using the same ethanol precipitation protocol described above. The purified pellet was dissolved in 20 µL of HI-DI formamide (Applied Biosystems, Foster City, CA, USA) and transferred to a 96-well plate. Capillary electrophoresis was performed on an ABI Prism 3500 Genetic Analyzer (Applied Biosystems, Foster City, CA, USA) according to manufacturer recommendations.

### 2.8. Genotypic and Mutational Analysis

Consensus sequence assembly from sequencing fragments was performed using “Unipro UGENE” v. 47 software [52]. Primary analysis of the obtained fragments was performed using the Nucleotide BLAST algorithm (https://blast.ncbi.nlm.nih.gov/Blast.cgi (accessed on 1 May 2026)) on the nucleotide sequences provided in the GenBank sequence database. The resulting sequences were aligned in the MEGA v.11 program using the MUSCLE algorithm [53]. HBV subgenotypes were determined by phylogenetic analysis of full-length HBV genome sequences, using reference sequences from GenBank representing major genotypes and subgenotypes. The phylogenetic tree was constructed using the neighbor-joining method. The significance of the tree was assessed using bootstrap analysis with 1000 replicates. The nucleotide sequences obtained were submitted to the HBVdb database (https://hbvdb.lyon.inserm.fr/HBVdb/HBVdbIndex (accessed on 11 May 2026)) [54] and the Genafor database (https://hbv.geno2pheno.org (accessed on 11 May 2026)) [55] to search for possible mutations. Protein amino acid sequences were determined by translating the corresponding nucleotide sequence according to the open reading frame. Amino acid substitutions were classified as immune escape mutations if they had been previously reported to reduce HBsAg antigenicity or impair anti-HBs binding, regardless of their frequency in the corresponding genotype. This approach reflects the fact that substitutions which are common in a given genotype as polymorphisms may nevertheless retain functional significance as escape mutations. Substitutions that were consensus residues in the corresponding subgenotype but lacked functional data were classified as genotype-associated polymorphisms.

### 2.9. Statistical Analysis

Statistical data processing was carried out using GraphPad Prism 5.0 (GraphPad Software, Inc.; USA, California San Diego). Key statistical results were independently verified using R software (version 4.3.2). Microsoft Excel (Microsoft Corp., USA, WA, Redmond) was used for data organization, table preparation, and graphical presentation. To assess statistical error, the exact Clopper–Pearson interval was applied. Results are presented with a 95% confidence interval (CI). For frequencies of 0%, the lower bound of the 95% CI was set to 0.0%. For frequencies of 100%, the upper bound was set to 100.0%, consistent with the properties of the exact binomial distribution. For assessing the significance of differences in quantitative data during paired comparisons, either Fisher’s exact test, or the chi-square test with Yates’ correction, was used depending on sample characteristics. A probability value of *p* < 0.05 was set as the threshold for statistical significance. Correlation analysis was conducted, taking into account compliance with parametric distribution, with calculation of Spearman rank correlation coefficients (rs). Differences were considered statistically significant when *p* < 0.05.

## 3. Results

### 3.1. Prevalence of Serological Markers and Profiles, Stratified by Sex and Age

The overall prevalence of HBV serological markers and the distribution of serological profiles in the study cohort (*n* = 874) are summarized in Table 2. Results from the total anti-HBc and anti-HBc IgG assays were concordant in all tested samples, indicating the absence of detectable anti-HBc IgM in the cohort. In the overall group, HBsAg prevalence was 2.06%, while anti-HBs IgG and anti-HBc IgG were detected in 76.54% and 28.95% of participants, respectively. Regarding serological profiles, the most common pattern was anti-HBs IgG only, observed in 53.2% of the cohort, followed by the combined presence of anti-HBc IgG and anti-HBs IgG in 22.88%. HBsAg positivity alone was rare (1.6%). Concurrent detection of all three markers (HBsAg, anti-HBc IgG, anti-HBs IgG) was observed in only 0.46% of the total group. Notably, the «anti-HBc only» profile was detected in 5.61% of the cohort, representing a group with serological evidence of HBV infection who would not be identified by HBsAg-based screening.

When stratified by sex, significant differences in marker prevalence were observed. The frequency of anti-HBs IgG was significantly higher in males (80.18%) than in females (70.52%; χ^2^ = 10.15, *p* = 0.0014). Similarly, anti-HBc IgG prevalence was significantly higher in males (33.94%) than in females (20.67%; χ^2^ = 16.94, *p* < 0.0001). Although no significant sex-based differences were found for the presence of anti-HBs IgG alone or anti-HBc IgG alone, the combined profile of “anti-HBc IgG and anti-HBs IgG” was significantly more frequent in males (27.16%) than in females (15.81%; χ^2^ = 14.34, *p* = 0.0002; OR = 1.99, 95% CI: 1.4–2.8).

**Figure 2:**
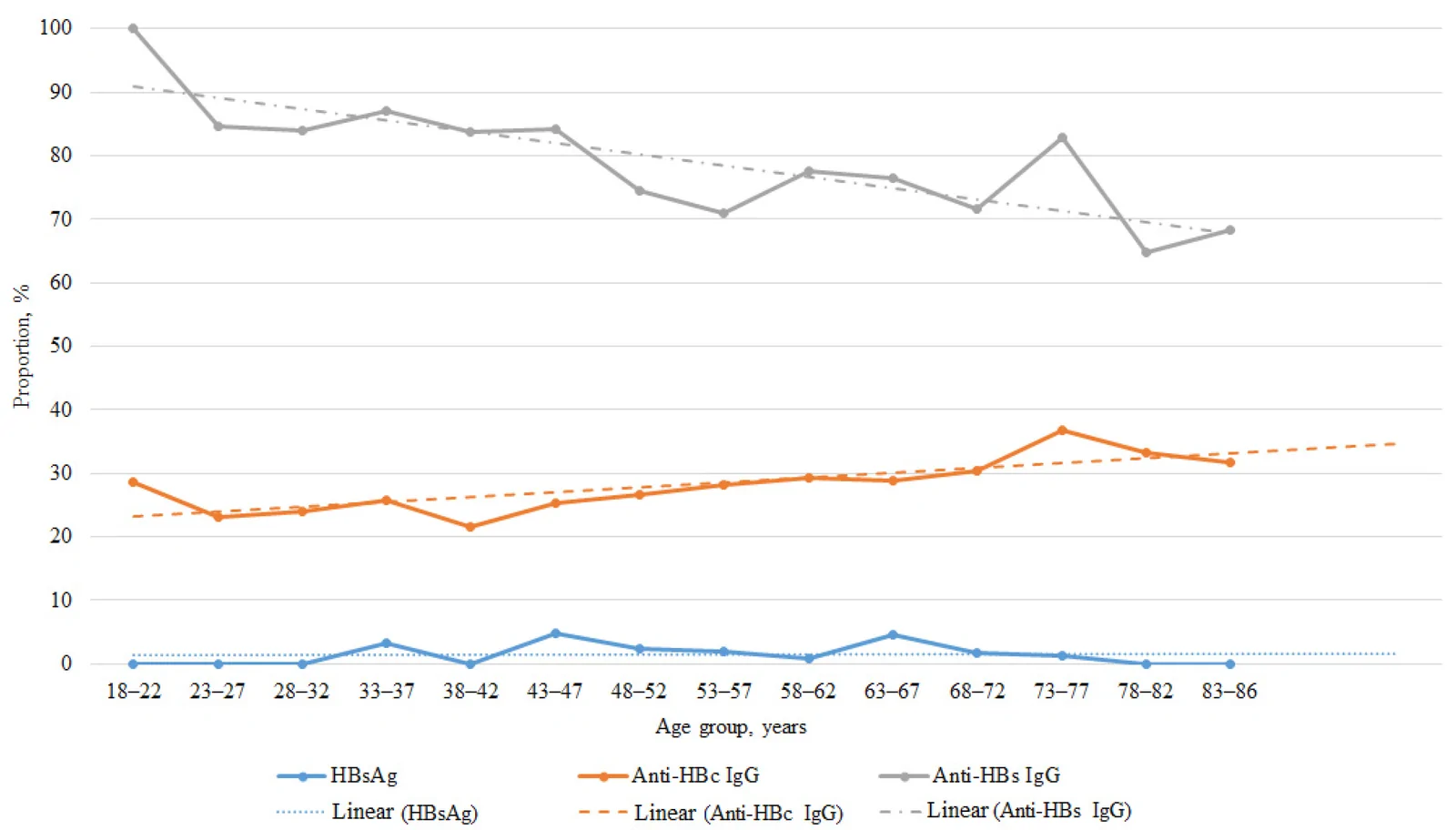
Correlation between age groups and prevalence of HBsAg, anti-HBc IgG, and anti-HBs IgG. Points represent observed prevalence for each age group. Lines connecting points indicate changes between consecutive age groups. Linear trends fitted by the least-squares method are shown for visualization purposes only and do not represent a separate regression analysis.

The prevalence of each serological marker and the distributions of serological profiles were further analyzed across age groups. Figure 2 provides a visualization of these marker trends. Detailed data are provided in Appendix A (Table A1 and Table A2).

Notably, a significant inverse correlation was observed between age and the prevalence of anti-HBs IgG (Spearman’s rs = −0.85, *p* = 0.0001), indicating a decline in this marker with advancing age. Conversely, the prevalence of anti-HBc IgG showed a significant positive correlation with age (Spearman’s rs = 0.8, *p* = 0.0003). No significant age-related trends were identified for the overall prevalence of HBsAg across the age groups.

### 3.2. Serological Marker and Profile Prevalence According to Dialysis Vintage and Underlying Kidney Disease

A progressive increase in the prevalence of anti-HBc IgG was observed with longer dialysis vintage: 3.8% in those with <1 year; 20.4% in the 1–5 years group; 43.1% in the >5–10 years group; and 31.0% in those with >10 years. Comparison between very disparate vintage groups (<1 year vs. >10 years) revealed a significantly higher prevalence of anti-HBc IgG in the latter group (Fisher’s exact test, *p* < 0.0001, OR = 11.25, 95% CI: 3.1–40.5). In contrast, anti-HBs IgG seroprevalence showed a downward trend with longer dialysis vintage, decreasing from 83.3% in the <1 year group to 69.0% in the >10 years group, although this difference did not reach significance (*p* = 0.06). No trend was observed for HBsAg prevalence across dialysis vintage groups (Table A3).

**Figure 3:**
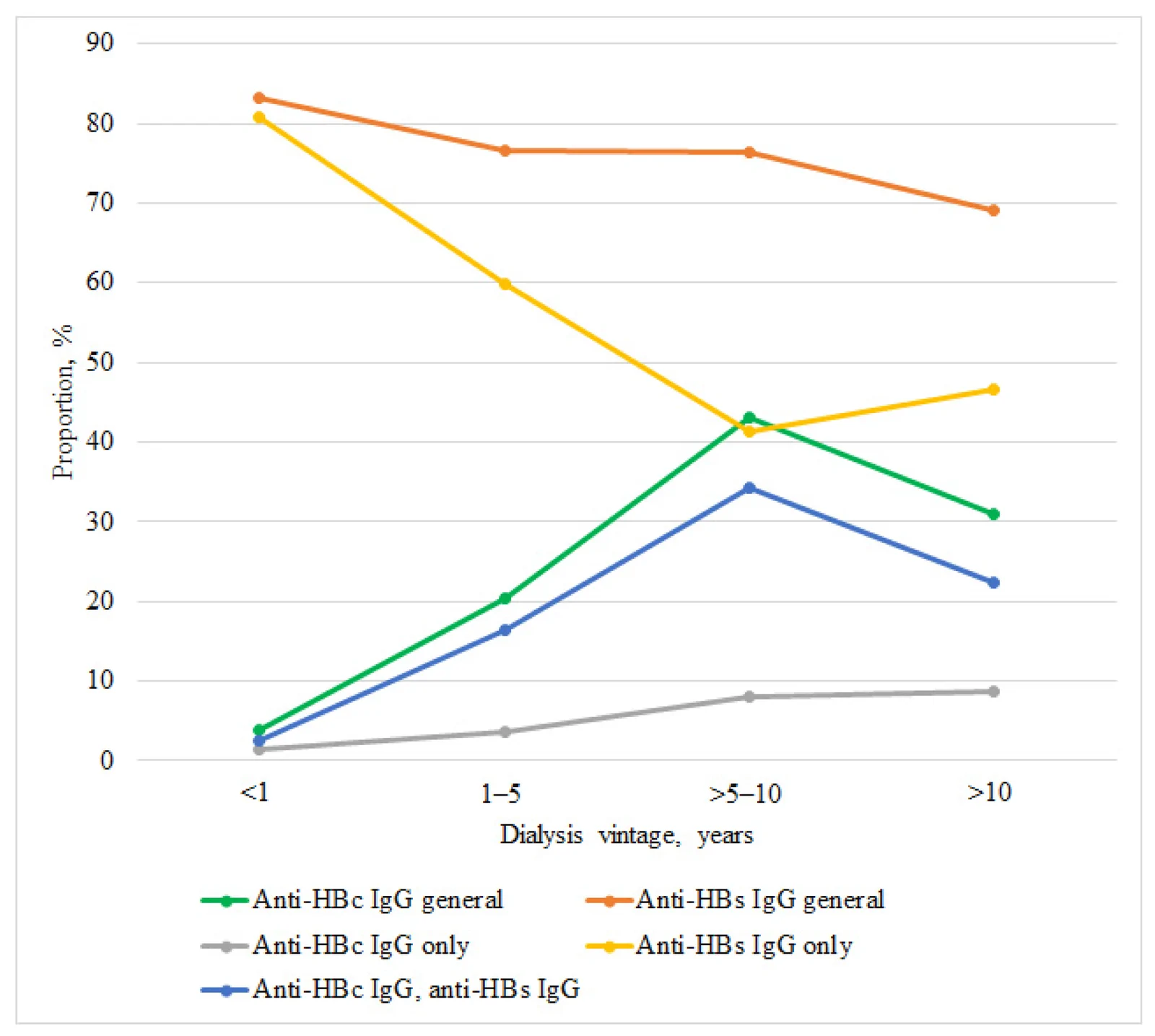
Trends in markers and selected profiles by dialysis vintage. Points represent observed prevalence for each dialysis vintage group (<1, 1–5, >5–10, >10 years). Lines connecting points indicate changes between consecutive groups.

The shifts in both marker prevalence and serological profile across dialysis vintage groups are visualized in Figure 3. The figure displays the trends for markers generally (anti-HBc IgG, anti-HBs IgG) and for their combinatorial IgG profiles (“anti-HBs only”, “anti-HBc only”, “anti-HBc/anti-HBs”).

Analysis of serological profiles revealed a substantial reorganization of immune status with longer dialysis duration. The proportion of patients with anti-HBs IgG only decreased progressively from 80.8% in the <1 year group to 46.6% in the >10 years group. A comparison between the most disparate groups revealed a significantly lower prevalence of this profile in the >10 years group compared to the <1 year group (χ^2^ = 15.9, *p* < 0.0001; OR = 0.20, 95% CI: 0.09–0.4).

The combined profile of “anti-HBc IgG and anti-HBs IgG” increased from 2.6% in the <1 year group to 22.4% in the >10 years group. There was a significantly higher prevalence in the >10 years group compared to the <1 year group (Fisher’s exact test, *p* = 0.0004; OR = 10.9, 95% CI: 2.4–50.9).

The anti-HBc IgG alone profile increased with longer dialysis vintage, rising from 1.3% in the <1 year group to 3.7% in the 1–5 years group, 8.1% in the >5–10 years group, and 8.6% in the >10 years group. Comparison between the most disparate groups (<1 year vs. >10 years) did not reveal a statistically significant difference (Fisher’s exact test, *p* = 0.078), likely due to the small number of positive cases in the relatively small groups. However, when comparing the 1–5 years and >5–10 years groups, a significantly higher prevalence was observed in the latter (χ^2^ = 5.6, *p* = 0.018; OR = 2.3, 95% CI: 1.2–4.4) (Table A4).

When stratified by the four etiological categories of ESRD (diabetes mellitus, arterial hypertension, primary kidney disease, other causes), no statistically significant differences were observed in the prevalence of any HBV serological marker (Table 3).

Similarly, the distribution of serological profiles was comparable across all disease categories, with anti-HBs IgG alone being the most frequent profile in each group, followed by the combined “anti-HBc IgG and anti-HBs IgG” profile (Table A5).

### 3.3. Viral DNA Seroprevalence Stratified by Age, Dialysis Vintage, and Renal Etiology

HBV DNA was detected in 32 out of 874 patients, yielding an overall prevalence of 3.7% (95% CI: 2.5–5.1). Among these, 18 patients were HBsAg-positive (chronic hepatitis B, CHB), corresponding to a prevalence of 2.1% (95% CI: 1.2–3.2), while 14 patients met the criteria for occult hepatitis B infection (OBI), with a prevalence of 1.6% (95% CI: 0.9–2.7).

No significant association with sex was found for either CHB or OBI (*p* > 0.05 for both). Analysis across age groups did not reveal any association with age for HBV DNA detection overall, nor for CHB or OBI separately (all *p* > 0.05, Table A6).

Stratification by dialysis vintage (Table A7) and by underlying cause of ESRD (Table A8) showed no statistically significant differences in HBV DNA, CHB, or OBI prevalence across any of the categories (all *p* > 0.05). Notably, no CHB cases were detected among patients with a dialysis vintage of <1 year or >10 years.

The serological profiles of the 32 patients with detectable HBV DNA are summarized in Table 4. Notably, the majority of CHB patients (77.8%) were seronegative for all tested Ab markers, whereas anti-HBs IgG only was the most common pattern (50.0%) in the OBI group.

Viral load was measured in all 32 patients with detectable HBV DNA. Two distinct patterns were observed based on HBsAg status. Among the 18 HBsAg-positive individuals, viral loads ranged from 7843 IU/mL to 64,870,000 IU/mL. Fourteen of these participants, all of whom were seronegative for anti-HBs and anti-HBc, had viral loads between 1,142,000 IU/mL and 64,870,000 IU/mL (mean ~12,416,000 IU/mL, median 7,965,000 IU/mL). The remaining four, who were positive for both anti-HBc and anti-HBs, had notably lower viral loads ranging from 7843 IU/mL to 94,612 IU/mL (mean 57,893 IU/mL, median 43,117 IU/mL).

In contrast, among the 14 individuals who tested negative for HBsAg (OBI group), viral load was uniformly low and did not exceed 150 IU/mL, ranging from 6.0 IU/mL (<LLOQ) to 148.3 IU/mL (mean 44.1 IU/mL, median 21.8 IU/mL). The highest viral load in the OBI cohort (148.3 IU/mL) was observed in a single patient with anti-HBc IgG only. Among the seven patients with anti-HBs IgG only, viral loads ranged from 6.0 (<LLOQ) to 43.7 IU/mL (median 14.2 IU/mL). The four patients with both anti-HBc and anti-HBs had loads between 23.8 and 138.1 IU/mL (median 76.2 IU/mL). The two seronegative OBI patients had detectable levels of 6.2 (<LLOQ) and 17.9 IU/mL, respectively. Notably, these values often fell below, or near, the lower limit of quantification of the commercial assay. Therefore, in accordance with Section 2.6, they should be interpreted as semi-quantitative estimates.

### 3.4. HBV Genotypic Distribution, Phylogenetic Analysis, and Clinically Relevant Mutations

**Figure 4:**
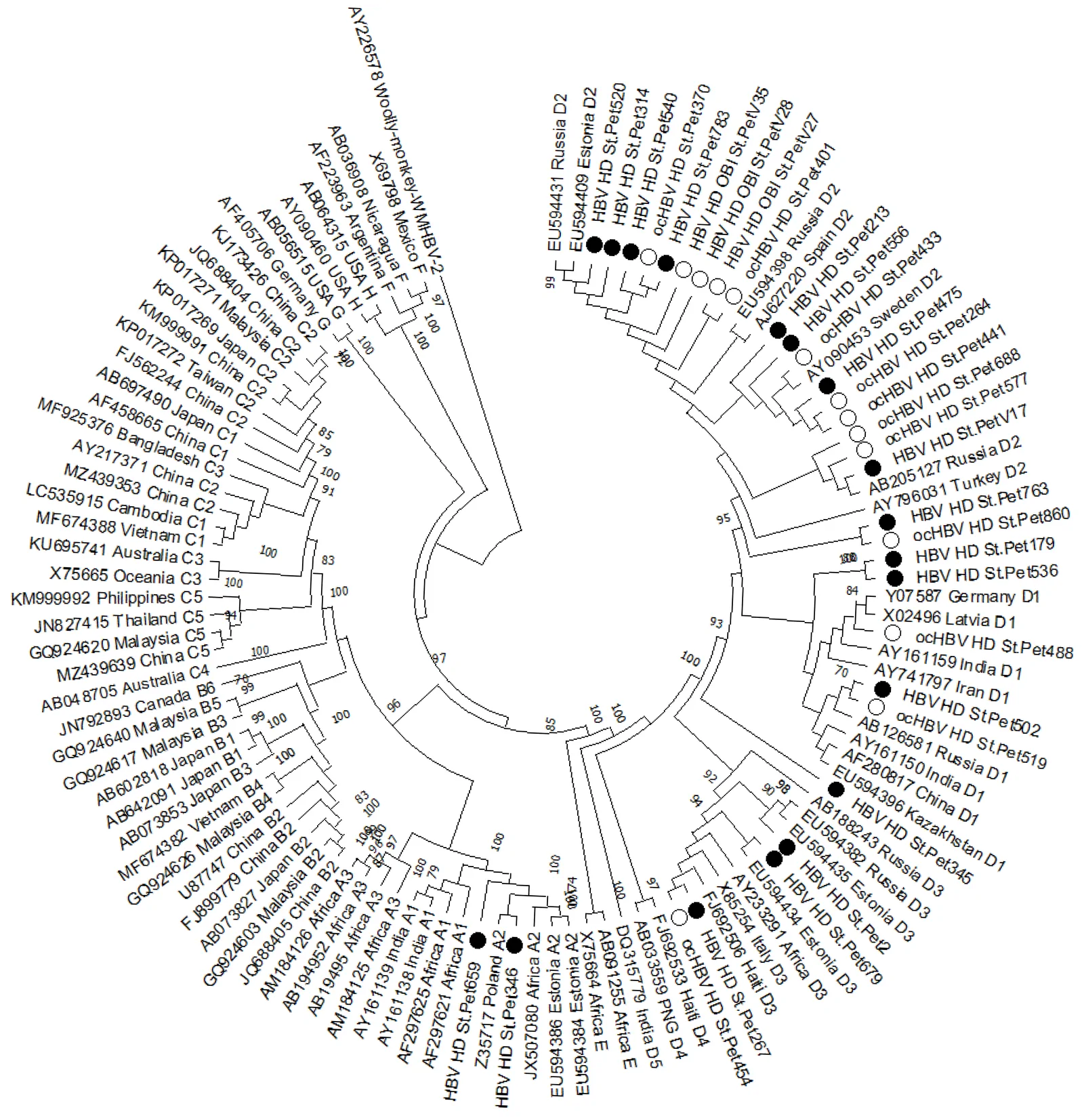
Phylogenetic analysis of HBV whole-genome nucleotide sequences isolated from patients on hemodialysis. The tree was constructed using the neighbor-joining (NJ) method based on the Kimura 2-parameter (K2P) model with 1000 bootstrap replicates. Bootstrap values ≥70% are shown at the nodes. Reference sequences available in GenBank were used for comparison and are designated with accession numbers indicating genotype and region of sample origin. The Woolly Monkey HBV nucleotide sequence (AY226578) was used as the outgroup. The samples studied in this work are indicated by black circles (HBsAg+) and white circles (HBsAg−).

The whole-genome nucleotide sequences of the identified HBV isolates have been submitted to GenBank under accession numbers OK998519-OK998523 and PX522122-PX522148. Phylogenetic analysis was performed on the obtained nucleotide sequences (Figure 4).

Among identified cases, genotype D was the most prevalent, accounting for 30 cases (93.8%; 95% CI: 79.2–99.2). This genotype was further stratified into subgenotypes D1 (8/32, 25%; 95% CI: 11.5–43.4), D2 (18/32, 56.3%; 95% CI: 37.7–73.6), and D3 (4/32, 12.5%; 95% CI: 3.5–29.0). Genotype A, specifically subgenotype A2, was identified in only two cases (6.3%; 95% CI: 0.8–20.8).

An analysis of OBI prevalence across the identified subgenotypes was performed. Among samples of subgenotype D3, OBI was confirmed in one case (1/4, 25%; 95% CI: 0.6–80.6). The highest observed prevalence was in subgenotype D2, with 10 cases (10/18, 55.6%; 95% CI: 30.8–78.5), followed by subgenotype D1 (3/8, 37.5%; 95% CI: 8.5–75.5). In contrast, OBI was not detected in the two samples belonging to subgenotype A2. However, due to the wide and overlapping confidence intervals, no statistically significant differences in OBI prevalence were established between the subgenotypes.

To further investigate potential associations, we examined the distribution of these subgenotypes across different disease groups and dialysis vintage categories. A comprehensive breakdown of these data, including the corresponding 95% confidence intervals, is provided in Table A9. Overall, no statistically significant differences were observed in subgenotype distribution with respect to these clinical and demographic parameters.

Drug resistance mutations (DRMs) were identified in two samples (2/32, 6.3%; 95% CI: 0.8–20.8). The L180M/M204V mutations, which confer resistance to lamivudine and telbivudine, were detected in one patient with OBI (1/14, 7.1%; 95% CI: 0.2–33.9). This patient belonged to disease group 2, had a dialysis vintage of >5–10 years, and was infected with HBV subgenotype D2. The triple mutation L180M/T184A/M204V (associated with lamivudine, telbivudine, and entecavir resistance) was found in one patient with CHB (1/18, 5.6%; 95% CI: 0.1–27.3). This patient belonged to disease group 1, had a dialysis vintage of 1–5 years, and was infected with HBV subgenotype D3. Due to the small number of DRM-positive cases, no statistical comparisons were performed. It should be noted that, due to the overlapping reading frames of the RT and S genes, the aforementioned mutations in the RT region may affect HBsAg protein antigenicity [56].

Immune escape mutations (IEMs) were detected in 50.0% of the study population (16/32; 95% CI: 31.9–68.1). A striking disparity was observed when stratifying by clinical status. IEM were present in 92.9% (13/14; 95% CI: 66.1–99.8) of OBI cases compared to only 16.7% (3/18; 95% CI: 3.6–41.4) of CHB cases (Fisher’s exact test, *p* < 0.0001). The odds of harboring IEM were significantly elevated in patients with OBI (OR = 65.0; 95% CI: 6.0–704.1), although the wide confidence interval reflects the limited sample size and underscores the need for validation in larger cohorts.

The frequencies of individual immune escape amino acid substitutions were further analyzed. The most frequently observed mutation was A128V, identified in 10 of 32 cases (31.3%), followed by R122K (5/32, 15.6%) and D144E (4/32, 12.5%). A comprehensive breakdown of all identified escape mutations, including frequencies and 95% confidence intervals for the total, CHB, and OBI groups, is provided in Table A10.

Precore mutations were identified in 8 of 32 patients (25.0%; 95% CI: 11.5–43.4). When stratified by clinical status, precore mutations were detected in two CHB patients (2/18, 11.1%; 95% CI: 1.4–34.7) and in six OBI patients (6/14, 42.9%; 95% CI: 17.7–71.1). Among the identified substitutions, the most frequently observed was W28 (including W28*, W28*Y, W28*W), found in 6 cases (6/32, 18.8%; 95% CI: 7.2–36.4), followed by G29D in 3 cases (3/32, 9.4%; 95% CI: 2.0–25.0). The W28* substitution corresponds to the G1896A nucleotide mutation, which creates a premature stop codon at amino acid position 28 of the precore protein. The designations W28*Y and W28*W represent mixed populations, in which both the stop codon (W28*) and an alternative amino acid (Y or W) are detected at the same position. Single occurrences were noted for C14S, L22M, and L24P (1/32, 3.1%; 95% CI: 0.1–16.2).

The classical double mutation A1762T/G1764A in the basal core promoter was detected in 8 cases (25.0%; 95% CI: 11.5–43.4), of which 5 occurred in CHB patients (5/18, 27.8%; 95% CI: 9.7–53.5) and 3 in OBI patients (3/14, 21.4%; 95% CI: 4.7–50.8).

Core region mutations were identified in 30 of 32 patients (93.7%; 95% CI: 79.2–99.2). The most frequently observed substitutions were N74V/G/T/A, N87S, and I97F, each detected in 29 cases (90.6%), followed by L116I in 28 cases (87.5%). Other recurrent mutations included T12S/N (7/32, 21.9%), E40D (6/32, 18.8%), A80T/I/G (5/32, 15.6%), and I59M/S/V (4/32, 12.5%). The remaining substitutions were observed in fewer than three cases each. A complete breakdown of all identified core region mutations, including their frequencies and 95% confidence intervals, is provided in Table A11.

No statistically significant differences were observed in the frequency of immune escape, precore, core promoter, or core region mutations when stratified by disease group or dialysis vintage (Fisher’s exact test, *p* > 0.05 for all comparisons).

## 4. Discussion

### 4.1. OBI Prevalence and Regional Context

In the present study, OBI prevalence (1.6%) falls within the lower end of the globally reported range (0–58%) [15] and is consistent with data from low-to-intermediate endemicity regions [57,58], but lower than reported in some Middle Eastern cohorts [44,59,60]. In this cohort, seronegative OBI accounted for 14.3% of all OBI cases. Farshadpour et al. (2023) found that 62% of their OBI patients were seronegative for all markers [59], while Samadi et al. (2020) reported that seronegative OBI was more common than Ab-seropositive OBI [44]. However, given the small number of OBI cases, these observations should be interpreted with caution. Since our assay is highly sensitive, this discrepancy more likely reflects an epidemiological transition in Russia driven by universal vaccination since the early 2000s [42] and centralized infection control in St. Petersburg dialysis centers. Genotype D predominance, including D2, D1, and D3, is consistent with the molecular epidemiology of HBV in Russia and neighboring regions [22,42,59,61].

### 4.2. Atypical Serological Profiles: Anti-HBc-Negative CHB and Anti-HBc-Only

Among the hemodialysis patients examined, we found an unexpectedly high proportion of HBsAg positivity in the absence of anti-HBc. This serological pattern is unusual in immunocompetent individuals, where anti-HBc appears during acute infection and persists for life [2,3]. This could suggest recent infection in these patients. However, this assumption is not supported by our data. First, no anti-HBc IgM was detected in any patient. Second, recent infection of such a large number of patients within a similar time frame would be expected to reflect an outbreak in a single healthcare facility. Our patients, however, were from different dialysis centers, and, more importantly, they carried different viral variants, including different subgenotypes. In contrast, patients with ESRD exhibit impaired cellular immunity, including reduced lymphoproliferative responses to mitogens, diminished T-cell function, and accelerated immune senescence [62,63]. This is consistent with the well-documented suboptimal serological response to HBV vaccination in hemodialysis patients [64], who are known to have uremia-associated immune dysfunction, including impaired T-cell responses and reduced antibody production, which may prevent anti-HBc development even in the presence of active viral replication [12,13,14]. Thus, in hemodialysis patients, the absence of anti-HBc cannot be interpreted as evidence against active HBV infection.

At the same time, the presence of «anti-HBc only» profile should not be overlooked. This profile warrants particular attention because it identifies patients with serological evidence of HBV infection who are not detected by HBsAg-based screening. Such patients remain at risk of HBV reactivation under immunosuppressive conditions, particularly after kidney transplantation [5,8]. Moreover, the absence of anti-HBs in these patients suggests an impaired humoral response, consistent with the uremia-associated immune dysfunction characteristic of hemodialysis patients [12,13,14].

### 4.3. Viral Load Patterns and Serological Correlates

A clear dichotomy in viral load was observed according to clinical status and serological profile. CHB patients generally had high viremia, whereas OBI patients had uniformly low viral loads. Within the CHB group, the highest levels were observed in patients seronegative for both anti-HBc and anti-HBs, whereas those positive for both markers had comparatively low loads, indicating more effective immune control of viral replication. Within the OBI group, a gradient in viral load was observed according to serological profile: the single patient with anti-HBc only had the highest level, those with combined anti-HBc/anti-HBs had intermediate levels, and patients with anti-HBs IgG only had low levels, while the two seronegative OBI patients had the lowest levels. This gradient suggests an association between residual serological reactivity and the level of residual viremia. These findings align with Sowole et al. (2015), who detected HBV DNA at similarly low levels (3–9 IU/mL) in their London cohort [57], and with the broader literature showing that OBI viral loads rarely exceed 200 IU/mL [2,3,7,25,59].

### 4.4. Risk Factors: Sex-Based Differences and Dialysis Vintage

Significant sex-based differences in HBV serological marker prevalence were observed. These findings are consistent with some reports [62] but not others [44]. These differences could potentially be explained by behavioral factors, including higher rates of high-risk practices in males [9,10], or by biological factors such as sex hormones influencing immune responses [63]. However, given the limited number of HBV DNA-positive cases, these differences should be interpreted with caution and warrant further investigation in larger cohorts.

A progressive increase in anti-HBc prevalence was observed with longer dialysis vintage, supporting the hypothesis that hemodialysis itself serves as a major risk factor for HBV acquisition [9,10]. This is consistent with previous systematic reviews identifying dialysis vintage ≥2 years as a significant risk factor [10] and with studies linking seroconversion to longer dialysis duration and multi-center attendance [62], highlighting the role of inter-facility patient transfer in HBV transmission. The parallel decline in the isolated anti-HBs IgG profile supports the concept of immune senescence in long-term hemodialysis patients [62,63].

No statistically significant association was observed between dialysis vintage and OBI prevalence, despite a clear progressive increase in anti-HBc seroprevalence with longer dialysis duration. This discrepancy may reflect the limited number of OBI cases and the low viral loads characteristic of OBI, which frequently fall below the limit of detection even with highly sensitive methods.

### 4.5. Immune Escape Mutations in OBI

The prevalence of immune escape mutations was significantly higher in OBI patients compared to CHB patients. This association provides molecular evidence that mutations in the surface protein, particularly within the “a” determinant of the major hydrophilic region (MHR, amino acids 124–147), may contribute to the occult phenotype by impairing HBsAg detection [29,30,31]. While this association has been suggested in smaller studies [17,35,59], our data support it specifically in hemodialysis patients, where viral genetic escape converges with uremia-associated immunosuppression [12,13].

The most frequent immune escape substitution was A128V, found in 31.3% of all patients and in 50% of OBI cases. Other common mutations included R122K (15.6% overall, 35.7% in OBI), D144E (12.5% overall, 28.6% in OBI), and S143P/L (9.4% overall, 21.4% in OBI). These mutations are located within or adjacent to the “a” determinant and have been previously described in OBI patients [17,32,33,34,35,36,37,38]. The classical immune escape mutation G145R, which is primarily known as a vaccine escape variant and can impair HBsAg detection by some immunoassays [38,39], was identified in only one patient (3.1%), an OBI case with a viral load of 43.7 IU/mL.

Some frequently observed genotype- or subtype-associated substitutions have also been reported to impair HBsAg detection and anti-HBs binding; we therefore retain them as functional escape mutations. A128V illustrates this: it is the consensus residue in subgenotype D2 but has been described as immune escape, associated with reduced HBsAg recognition and occult infection [65,66,67,68]. The same applies to R122K (which determines the d/y serotype) and T127P (characteristic of D2), both of which impair HBsAg recognition and have been associated with vaccine escape [2,18]. The low frequency of G145R in this cohort suggests that other mutations may be more relevant to OBI pathogenesis in the Russian hemodialysis population.

Recent mechanistic work showed that the M133S mutant induces ER stress and mitophagy, impairing HBsAg secretion [36]—a framework likely applicable to other «a» determinant mutations that alter conformational epitopes required for antibody binding [17,35].

### 4.6. Core, Precore, and Basal Core Promoter Mutations

Core region mutations were present in nearly all patients, with N74V/G/T/A, N87S, I97F, and L116I predominating. These have been described in both OBI and CHB and may contribute to altered capsid stability and immune evasion [40,51], potentially through changes in T-cell epitopes of the core protein (HBcAg), a major target of cytotoxic T lymphocytes [40]—particularly relevant in hemodialysis patients, where T-cell dysfunction is a known feature of uremia [12,13,14]. The near-universal presence of core mutations in this cohort, regardless of clinical status, suggests that these mutations may be associated with chronic HBV infection in hemodialysis patients.

Precore mutations were more frequent in OBI than in CHB patients, with W28 being the most common. Stop-codon mutations here prevent HBeAg expression, which has been associated with reduced viral replication and immune evasion [40]. HBeAg is a tolerogen that helps the virus evade the host immune response by inducing T-cell tolerance [40]; its loss may facilitate immune clearance in some patients, leading to the low-level persistent infection characteristic of OBI. In immunosuppressed hemodialysis patients, however, the loss of HBeAg may not result in viral clearance, but rather in a state of low-level replication that escapes immune detection.

The classical double mutation A1762T/G1764A in the basal core promoter was found in a similar proportion of CHB and OBI patients. This mutation is associated with reduced HBeAg synthesis, increased viral replication, more severe liver disease, and an increased risk of hepatocellular carcinoma [40].

### 4.7. Drug Resistance Mutations

Drug resistance mutations were identified in two patients without documented nucleos(t)ide analogue therapy—L180M/M204V in an OBI patient and L180M/T184A/M204V in a CHB patient. These could have been acquired before or during dialysis, including through horizontal transmission within dialysis settings, as previously suggested for hemodialysis patients without documented antiviral exposure [69].

Because the RT and S genes overlap, mutations in one may affect the other: RT mutations can alter HBsAg antigenicity [56], and immune pressure on the S gene may inadvertently select for drug-resistant RT variants even without antiviral therapy. This underscores the need for resistance surveillance in hemodialysis populations.

### 4.8. Limitations

Several limitations of this study should be acknowledged. The cross-sectional design precludes assessment of temporal changes in HBV marker prevalence or viral dynamics. The absence of liver biopsy specimens, the gold standard for OBI diagnosis, means intrahepatic HBV DNA could not be confirmed, although detection in multiple plasma aliquots provides strong evidence of infection. The limited number of OBI cases (*n* = 14) reduced statistical power for subgroup analyses. The viral load values in OBI patients, which often fell below the lower limit of quantification of the commercial assay (15 IU/mL), should be interpreted as semi-quantitative estimates expressed in IU/mL, rather than absolute quantitative measurements. This is a common limitation in OBI studies, where low-level viremia frequently requires modified extraction protocols. In addition, samples with HBV DNA concentrations below 5 IU/mL, the lower limit of detection of the in-house assay, cannot be detected. Importantly, this does not affect the main qualitative findings of our study, insofar as OBI cases were confirmed by detection of HBV DNA in two independent extraction and amplification rounds. Since only HBsAg, anti-HBc, and anti-HBs IgG were tested, «seronegativity» in this study refers to the absence of these three markers only; the presence of other HBV serological markers (HBeAg, anti-HBe) cannot be excluded. HBsAg concentrations below 0.01 IU/mL, the analytical sensitivity of the conventional assays used, could not be detected. The study was conducted in a single geographic region, and findings may not be generalizable to other populations. Finally, HBV DNA was measured on a single occasion, and transient viremia below the detection limit cannot be excluded.

## 5. Conclusions

This study assesses the prevalence of HBV infection in a cohort of 874 hemodialysis patients in St. Petersburg, Russia. Molecular analysis was performed on the 32 individuals with detectable HBV DNA. OBI was identified in 1.6% and overt CHB in 2.1% of patients, with genotype D predominating (93.8%). Immune escape mutations were significantly more prevalent in OBI patients than in CHB patients. Seronegative OBI cases show that serological testing alone is insufficient to identify all HBV-infected hemodialysis patients. A gradient in viral load according to serological profile suggests an association between serological status and residual viremia. The near-universal presence of core region mutations suggests that these mutations may be associated with chronic HBV infection in hemodialysis patients. The presence of drug resistance mutations and the progressive increase in HBV exposure with longer dialysis vintage underscore the need for molecular surveillance. These findings suggest that HBV DNA screening may warrant consideration in HD patients, regardless of serological profile. However, the clinical outcomes, transmission risk, and cost-effectiveness of such an approach remain to be evaluated in prospective studies.

## Figures and Tables

**Table 1:** Primer and probe sequences used for the detection of HBV DNA in plasma with low viral load by second-round, nested real-time PCR.

Primer/Probe Name	Sequence (5′ → 3′)
HBV SrtF2	5′-CACCTGTATTCCCATCCCATC-3′
HBV SrtR2	5′-AGCCCTACGAACCACTGAACA-3′
HBV Srt-Z	5′-FAM-AAACGGACTGAGGCCCACTCCCA-BHQ1/RTQ1-3′
HBV XrtF2	5′-GTCTGTGCCTTCTCATCTGCC-3′
HBV XrtRd2	5′-GTCGGTCGTTGACATTGCAG-3′
HBV Xrt-Z	5′-ROX-TGTGCACTTCGCTTCACCTCTGC-BHQ2/RTQ2-3′
HPRT F	5′-CTTGCTCGAGATGTGATGAAGG-3′
HPRT R	5′-CAGCAGGTCAGCAAAGAATTTATAG-3′
HPRT-Z	5′-Cy5-ATCACATTGTAGCCCTCTGTGTGCTCAAGG-BHQ2/RTQ2-3′

**Table 2:** HBV serological marker prevalence and distribution in the examined group (HBsAg, anti-HBc IgG, anti-HBs IgG).

HBV Serological Marker (Prevalence)	Number in the Overall Group (*n* = 874), Share of the Group	Number in the Male Group (*n* = 545), Share of the Male Group	Number in the Female Group (*n* = 329), Share of the Female Group
	*n* (%, 95% Confidence Interval)	*n* (%, 95% Confidence Interval)	*n* (%, 95% Confidence Interval)
HBsAg	18 (2.06%, CI: 1.23–3.24)	12 (2.20%, CI: 1.14–3.81)	6 (1.82%, CI: 0.67–3.93)
Anti-HBs IgG	669 (76.54%, CI: 73.59–79.32)	437 (80.18%, CI: 76.59–83.45)	232 (70.52%, CI: 65.52–75.39)
Anti-HBc IgG	253 (28.95%, CI: 25.96–32.08)	185 (33.94%, CI: 29.97–38.09)	68 (20.67%, CI: 16.42–25.45)
HBV Serological Profile (distribution)			
HBsAg only	14 (1.60%, CI: 0.88–2.67)	10 (1.83%, CI: 0.88–3.35)	4 (1.22%, CI: 0.33–3.08)
Anti-HBs IgG only	465 (53.20%, CI: 49.83–56.55)	287 (52.66%, CI: 48.37–56.92)	178 (54.10%, CI: 48.55–59.58)
Anti-HBc IgG only	49 (5.61%, CI: 4.18–7.34)	35 (6.42%, CI: 4.51–8.82)	14 (4.26%, CI: 2.35–7.04)
Anti-HBc IgG, anti-HBs IgG	200 (22.88%, CI: 20.14–25.81)	148 (27.16%, CI: 23.46–31.1)	52 (15.81%, CI: 12.04–20.2)
HBsAg, anti-HBc IgG, anti-HBs IgG	4 (0.46%, CI: 0.12–1.17)	2 (0.37%, CI: 0.04–1.32)	2 (0.61%, CI: 0.07–2.18)
Seronegative	142 (16.25%, CI: 13.86–18.86)	63 (11.56%, CI: 9.0–14.55)	79 (24.01%, CI: 19.5–29.0)

Note: HBsAg—hepatitis B surface antigen; anti-HBs IgG—IgG antibodies to HBsAg; anti-HBc IgG—IgG antibodies to hepatitis B core antigen; CI—confidence interval.

**Table 3:** Serological marker prevalence by underlying cause of end-stage renal disease.

Disease Group	*N*	HBsAg			Anti-HBc IgG			Anti-HBs IgG		
		*n*	%	95% CI	*n*	%	95% CI	*n*	%	95% CI
1	343	7	2	0.8–4.2	100	29.2	24.4–34.3	265	77.3	72.5–81.6
2	219	5	2.3	0.7–5.2	65	29.7	23.7–36.2	175	79.9	74.0–85.0
3	194	4	2.1	0.6–5.2	59	30.4	24.0–37.4	144	74.2	67.5–80.2
4	118	2	1.7	0.2–6.0	29	24.6	17.1–33.4	85	72	63.0–79.9

Note: HBsAg—hepatitis B surface antigen; anti-HBs IgG—IgG antibodies to HBsAg; anti-HBc IgG—IgG antibodies to hepatitis B core antigen; CI—confidence interval.

**Table 4:** Serological profiles of patients with detectable HBV DNA, stratified by HBsAg status (CHB vs. OBI).

HBV Serological Profile	CHB (*n* = 18)			OBI (*n* = 14)		
	*n*	%	95% CI	*n*	%	95% CI
Anti-HBs IgG only	0	0	0.0–18.5	7	50	23.0–77.0
Anti-HBc IgG only	0	0	0.0–18.5	1	7.1	0.2–33.9
Anti-HBc IgG, anti-HBs IgG	4	22.2	6.4–47.6	4	28.6	8.4–58.1
Seronegative for all tested Ab markers	14	77.8	52.4–93.6	2	14.3	1.8–42.8

Note: CHB—chronic hepatitis B; OBI—occult hepatitis B infection; anti-HBs IgG—IgG antibodies to HBsAg; anti-HBc IgG—IgG antibodies to hepatitis B core antigen; Ab—antibody; CI—confidence interval.

## Data Availability

The original contributions presented in this study are included in the article and Supplementary Materials. Further inquiries can be directed to the corresponding author.

## Supplementary Materials

The following supporting information can be downloaded at: https://www.mdpi.com/article/10.3390/diagnostics16213588/s1, Table S1: Thermal cycling conditions for the first round of nested PCR; Table S2: Thermal cycling conditions for the second round of nested PCR.

## Appendix A

**Table A1:** HBV serological marker prevalence by age group.

Age Group, Years	*n*	HBsAg			Anti-HBc IgG			Anti-HBs IgG		
		*n*	%	95% CI	*n*	%	95% CI	*n*	%	95% CI
18–22	7	0	0	0.0–41.0	2	28.6	3.7–71.0	7	100	59.0–100
23–27	13	0	0	0.0–24.7	3	23.1	5.0–53.8	11	84.6	54.6–98.1
28–32	25	0	0	0.0–13.7	6	24	9.4–45.1	21	84	63.9–95.5
33–37	31	1	3.2	0.1–16.7	8	25.8	11.9–44.6	27	87.1	70.2–96.4
38–42	37	0	0	0.0–9.5	8	21.6	9.8–38.2	31	83.8	68.0–93.8
43–47	63	3	4.8	1.0–13.3	16	25.4	15.3–37.9	53	84.1	72.7–92.1
48–52	86	2	2.3	0.3–8.1	23	26.7	17.8–37.4	64	74.4	63.9–83.2
53–57	103	2	1.9	0.2–6.8	29	28.2	19.7–37.9	73	70.9	61.1–79.4
58–62	116	1	0.9	0.0–4.7	34	29.3	21.2–38.5	90	77.6	68.9–84.8
63–67	132	6	4.5	1.7–9.6	38	28.8	21.2–37.3	101	76.5	68.4–83.5
68–72	109	2	1.8	0.2–6.5	33	30.3	21.8–39.8	78	71.6	62.1–79.8
73–77	76	1	1.3	0.0–7.1	28	36.8	26.1–48.7	63	82.9	72.5–90.6
78–82	54	0	0	0.0–6.6	18	33.3	21.1–47.5	35	64.8	50.6–77.3
83–86	22	0	0	0.0–15.4	7	31.8	13.9–54.9	15	68.2	45.1–86.1

Note: HBsAg—hepatitis B surface antigen; anti-HBs IgG—IgG antibodies to HBsAg; anti-HBc IgG—IgG antibodies to hepatitis B core antigen; CI—confidence interval.

**Table A2:** HBV serological profile distribution by age group.

Age Group, Years	*n*	HBsAg Only			Anti-HBc IgG Only			Anti-HBs IgG Only			Anti-HBc IgG, Anti-HBs IgG			HBsAg, Anti-HBc IgG, Anti-HBs IgG		
		*n*	%	95% CI	*n*	%	95% CI	*n*	%	95% CI	*n*	%	95% CI	*n*	%	95% CI
18–22	7	0	0	0.0–41.0	0	0	0.0–41.0	5	71.4	29.0–96.3	2	28.6	3.7–71.0	0	0	0.0–41.0
23–27	13	0	0	0.0–24.7	1	7.7	0.2–36.0	9	69.2	38.6–90.9	2	15.4	1.9–45.4	0	0	0.0–24.7
28–32	25	0	0	0.0–13.7	0	0	0.0–13.7	15	60	38.7–78.9	6	24	9.4–45.1	0	0	0.0–13.7
33–37	31	1	3.2	0.1–16.7	1	3.2	0.1–16.7	20	64.5	45.4–80.8	7	22.6	9.6–41.1	0	0	0.0–11.2
38–42	37	0	0	0.0–9.5	1	2.7	0.1–14.2	24	64.9	47.5–79.8	7	18.9	8.0–35.2	0	0	0.0–9.5
43–47	63	3	4.8	1.0–13.3	1	1.6	0.0–8.5	38	60.3	47.2–72.4	15	23.8	14.0–36.2	0	0	0.0–5.7
48–52	86	1	1.2	0.0–6.3	5	5.8	1.9–13.0	46	53.5	42.4–64.3	17	19.8	12.0–29.8	1	1.2	0.0–6.3
53–57	103	1	1	0.0–5.3	8	7.8	3.4–14.7	52	50.5	40.5–60.5	20	19.4	12.3–28.4	1	1	0.0–5.3
58–62	116	1	0.9	0.0–4.7	9	7.8	3.6–14.2	65	56	46.5–65.2	25	21.6	14.5–30.1	0	0	0.0–3.1
63–67	132	5	3.8	1.2–8.6	3	2.3	0.5–6.5	66	50	41.2–58.8	34	25.8	18.5–34.1	1	0.8	0.0–4.1
68–72	109	2	1.8	0.2–6.5	9	8.3	3.8–15.1	54	49.5	39.8–59.3	24	22	14.6–31.0	0	0	0.0–3.3
73–77	76	0	0	0.0–4.7	5	6.6	2.2–14.7	40	52.6	40.8–64.2	22	28.9	19.1–40.5	1	1.3	0.0–7.1
78–82	54	0	0	0.0–6.6	4	7.4	2.1–17.9	21	38.9	25.9–53.1	14	25.9	15.0–39.7	0	0	0.0–6.6
83–86	22	0	0	0.0–15.4	2	9.1	1.1–29.2	10	45.5	24.4–67.8	5	22.7	7.8–45.4	0	0	0.0–15.4

Note: HBsAg—hepatitis B surface antigen; anti-HBs IgG—IgG antibodies to HBsAg; anti-HBc IgG—IgG antibodies to hepatitis B core antigen; CI—confidence interval.

**Table A3:** Prevalence of HBV serological markers by dialysis vintage.

Dialysis Vintage, Years	*n*	HBsAg			Anti-HBc IgG			Anti-HBs IgG		
		*n*	%	95% CI	*n*	%	95% CI	*n*	%	95% CI
<1	78	0	0	0.0–4.6	3	3.8	0.8–10.8	65	83.3	73.2–90.8
1–5	378	6	1.6	0.6–3.4	77	20.4	16.4–24.8	289	76.5	71.8–80.6
>5–10	360	12	3.3	1.7–5.8	155	43.1	37.9–48.3	275	76.4	71.7–80.7
>10	58	0	0	0.0–6.2	18	31	19.5–44.5	40	69	55.5–80.5

Note: HBsAg—hepatitis B surface antigen; anti-HBs IgG—IgG antibodies to HBsAg; anti-HBc IgG—IgG antibodies to hepatitis B core antigen; CI—confidence interval.

**Table A4:** Distribution of HBV serological profiles by dialysis vintage.

Dialysis Vintage, Years	*n*	HBsAg Only			Anti-HBc IgG Only			Anti-HBs IgG Only			Anti-HBc IgG, Anti-HBs IgG			HBsAg, Anti-HBc IgG, Anti-HBs IgG		
		*n*	%	95% CI	*n*	%	95% CI	*n*	%	95% CI	*n*	%	95% CI	*n*	%	95% CI
<1	78	0	0	0.0–4.6	1	1.3	0.0–6.9	63	80.8	70.3–88.8	2	2.6	0.3–9.0	0	0	0.0–4.6
1–5	378	5	1.3	0.4–3.1	14	3.7	2.0–6.1	226	59.8	54.7–64.8	62	16.4	12.8–20.5	1	0.3	0.0–1.5
>5–10	360	9	2.5	1.1–4.7	29	8.1	5.5–11.4	149	41.4	36.3–46.7	123	34.2	29.3–39.3	3	0.8	0.2–2.4
>10	58	0	0	0.0–6.2	5	8.6	2.9–19.0	27	46.6	33.3–60.1	13	22.4	12.5–35.3	0	0	0.0–6.2

**Table A5:** Distribution of HBV serological profiles by ESRD etiology.

Disease Group	*n*	HBsAg Only			Anti-HBc IgG Only			Anti-HBs IgG Only			Anti-HBc IgG, Anti-HBs IgG			HBsAg, Anti-HBc IgG, Anti-HBs IgG		
		*n*	%	95% CI	*n*	%	95% CI	*n*	%	95% CI	*n*	%	95% CI	*n*	%	95% CI
1	343	3	0.9	0.2–2.5	21	6.1	3.8–9.2	186	54.2	48.8–59.6	75	21.9	17.6–26.6	4	1.2	0.3–3.0
2	219	5	2.3	0.7–5.2	10	4.6	2.2–8.2	120	54.8	47.9–61.5	55	25.1	19.5–31.4	0	0	0.0–1.7
3	194	4	2.1	0.6–5.2	13	6.7	3.6–11.2	98	50.5	43.3–57.8	46	23.7	17.9–30.3	0	0	0.0–1.9
4	118	2	1.7	0.2–6.0	5	4.2	1.4–9.6	61	51.7	42.3–61.0	24	20.3	13.5–28.7	0	0	0.0–3.1

Note: HBsAg—hepatitis B surface antigen; anti-HBs IgG—IgG antibodies to HBsAg; anti-HBc IgG—IgG antibodies to hepatitis B core antigen; CI—confidence interval.

**Table A6:** Prevalence of HBV DNA, CHB, and OBI by age group.

Age Group, Years	*n*	HBV DNA			CHB			OBI		
		*n*	%	95% CI	*n*	%	95% CI	*n*	%	95% CI
18–22	7	0	0	0.0–41.0	0	0	0.0–41.0	0	0	0.0–41.0
23–27	13	0	0	0.0–24.7	0	0	0.0–24.7	0	0	0.0–24.7
28–32	25	2	8	1.0–26.0	0	0	0.0–13.7	2	8	1.0–26.0
33–37	31	2	6.5	0.8–21.4	1	3.2	0.1–16.7	1	3.2	0.1–16.7
38–42	37	1	2.7	0.1–14.2	0	0	0.0–9.5	1	2.7	0.1–14.2
43–47	63	3	4.8	1.0–13.3	3	4.8	1.0–13.3	0	0	0.0–5.7
48–52	86	2	2.3	0.3–8.1	2	2.3	0.3–8.1	0	0	0.0–4.2
53–57	103	2	1.9	0.2–6.8	2	1.9	0.2–6.8	0	0	0.0–3.5
58–62	116	4	3.4	0.9–8.6	1	0.9	0.0–4.7	3	2.6	0.5–7.4
63–67	132	6	4.5	1.7–9.6	6	4.5	1.7–9.6	0	0	0.0–2.8
68–72	109	4	3.7	1.0–9.1	2	1.8	0.2–6.5	2	1.8	0.2–6.5
73–77	76	3	3.9	0.8–11.1	1	1.3	0.0–7.1	2	2.6	0.3–9.2
78–82	54	2	3.7	0.5–12.7	0	0	0.0–6.6	2	3.7	0.5–12.7
83–86	22	1	4.5	0.1–22.8	0	0	0.0–15.4	1	4.5	0.1–22.8

Note: HBV—hepatitis B virus; CHB—chronic hepatitis B; OBI—occult hepatitis B infection; CI—confidence interval.

**Table A7:** Prevalence of HBV DNA, CHB, and OBI by dialysis vintage.

Dialysis Vintage, Years	*n*	HBV DNA			CHB			OBI		
		*n*	%	95% CI	*n*	%	95% CI	*n*	%	95% CI
<1	78	1	1.3	0.0–6.9	0	0	0.0–4.6	1	1.3	0.0–6.9
1–5	378	11	2.9	1.5–5.1	6	1.6	0.6–3.4	5	1.3	0.4–3.1
>5–10	360	19	5.3	3.2–8.1	12	3.3	1.7–5.8	7	1.9	0.8–4.0
>10	58	1	1.7	0.0–9.2	0	0	0.0–6.2	1	1.7	0.0–9.2

Note: HBV—hepatitis B virus; CHB—chronic hepatitis B; OBI—occult hepatitis B infection; CI—confidence interval.

**Table A8:** Prevalence of HBV DNA, CHB, and OBI by ESRD etiology.

Disease Group	*n*	HBV DNA			CHB			OBI		
		*n*	%	95% CI	*n*	%	95% CI	*n*	%	95% CI
1	343	12	3.5	1.8–6.0	7	2	0.8–4.2	5	1.5	0.5–3.4
2	219	6	2.7	1.0–5.9	5	2.3	0.7–5.2	1	0.5	0.0–2.5
3	194	10	5.2	2.5–9.3	4	2.1	0.6–5.2	6	3.1	1.1–6.6
4	118	4	3.4	0.9–8.5	2	1.7	0.2–6.0	2	1.7	0.2–6.0

Note: HBV—hepatitis B virus; CHB—chronic hepatitis B; OBI—occult hepatitis B infection; CI—confidence interval.

**Table A9:** Distribution of disease groups and dialysis vintage categories by viral subgenotype.

Factor		D1 (*n* = 8)			D2 (*n* = 18)			D3 (*n* = 4)			A2 (*n* = 2)		
		*n*	%	95% CI	*n*	%	95% CI	*n*	%	95% CI	*n*	%	95% CI
Disease group	1	3	37.5	8.5–75.5	6	33.3	13.3–59.0	3	75	19.4–99.4	0	0	0.0–84.2
	2	1	12.5	0.3–52.7	3	16.7	3.6–41.4	0	0	0.0–60.2	2	100	15.8–100
	3	4	50	15.7–84.3	5	27.8	9.7–53.5	1	25	0.6–80.6	0	0	0.0–84.2
	4	0	0	0.0–36.9	4	22.2	6.4–47.6	0	0	0.0–60.2	0	0	0.0–84.2
Dialysis vintage, years	<1	0	0	0.0–36.9	1	5.6	0.1–27.3	0	0	0.0–60.2	0	0	0.0–84.2
	1–5	5	62.5	24.5–91.5	5	27.8	9.7–53.5	1	25	0.6–80.6	0	0	0.0–84.2
	>5–10	3	37.5	8.5–75.5	11	61.1	35.7–82.7	3	75	19.4–99.4	2	100	15.8–100
	>10	0	0	0.0–36.9	1	5.6	0.1–27.3	0	0	0.0–60.2	0	0	0.0–84.2

Note: CI—confidence interval.

**Table A10:** Frequencies of Individual Immune Escape Amino Acid Substitutions in the Total, CHB, and OBI Groups.

Amino Acid Substitution	Frequency of Occurrence in the Group (*n* = 32)		Frequency of Occurrence in the CHB Group (*n* = 18)		Frequency of Occurrence in the OBI Group (*n* = 14)	
	abs., *n*	%, 95% CI	abs., *n*	%, 95% CI	abs., *n*	%, 95% CI
Y100C	2	6.3% (0.8–20.8)	0	0.0% (0.0–18.5)	2	14.3% (1.8–42.8)
L109R	1	3.1% (0.1–16.2)	0	0.0% (0.0–18.5)	1	7.1% (0.2–33.9)
R122K	5	15.6% (5.3–32.8)	0	0.0% (0.0–18.5)	5	35.7% (12.8–64.9)
A128V	10	31.3% (16.1–50.0)	3	16.7% (3.6–41.4)	7	50.0% (23.0–77.0)
Q129R/H	2	6.3% (0.8–20.8)	0	0.0% (0.0–18.5)	2	14.3% (1.8–42.8)
G130R	2	6.3% (0.8–20.8)	0	0.0% (0.0–18.5)	2	14.3% (1.8–42.8)
C137Y/W	2	6.3% (0.8–20.8)	0	0.0% (0.0–18.5)	2	14.3% (1.8–42.8)
S143P/L	3	9.4% (2.0–25.0)	0	0.0% (0.0–18.5)	3	21.4% (4.7–50.8)
D144E	4	12.5% (3.5–29.0)	0	0.0% (0.0–18.5)	4	28.6% (8.4–58.1)
G145R	1	3.1% (0.1–16.2%)	0	0.0% (0.0–18.5)	1	7.1% (0.2–33.9)

Note: CHB—chronic hepatitis B; OBI—occult hepatitis B infection; CI—confidence interval.

**Table A11:** Frequencies of Identified Core Region Amino Acid Substitutions.

Amino Acid Substitution	Frequency of Occurrence in the Group (*N* = 32)			
	abs., *n*	%	95% CI−	95% CI+
T12S/N	7	21.9	9.3	40
L16I	2	6.3	0.8	20.8
S21Q	1	3.1	0.1	16.2
V27L	1	3.1	0.1	16.2
A34T	2	6.3	0.8	20.8
E40D	6	18.8	7.2	36.4
S49T	2	6.3	0.8	20.8
P50H	1	3.1	0.1	16.2
I59M/S/V	4	12.5	3.5	29
M66R	2	6.3	0.8	20.8
T67N	1	3.1	0.1	16.2
N74V/G/T/A	29	90.6	75	98
P79Q	3	9.4	2	25
A80T/I/G	5	15.6	5.3	32.8
V86IV	2	6.3	0.8	20.8
N87S	29	90.6	75	98
N92H	3	9.4	2	25
M93V/L	2	6.3	0.8	20.8
L95I	1	3.1	0.1	16.2
K96N	1	3.1	0.1	16.2
I97F	29	90.6	75	98
I105V	2	6.3	0.8	20.8
E113D	1	3.1	0.1	16.2
L116I	28	87.5	71	96.5
P130Q	1	3.1	0.1	16.2
T142I	1	3.1	0.1	16.2
L143I	2	6.3	0.8	20.8
R156G	1	3.1	0.1	16.2
S178T	1	3.1	0.1	16.2
Q179K	3	9.4	2	25
E182G	1	3.1	0.1	16.2

Note: CHB—chronic hepatitis B; OBI—occult hepatitis B infection; CI—confidence interval.

## References

[1] World Health Organization (2024). Implementing the Global Health Sector Strategies on HIV, Viral Hepatitis and Sexually Transmitted Infections, 2022–2030: Report on Progress and Gaps 2024.

[2] Raimondo G., Allain J.P., Brunetto M.R., Buendia M.A., Chen D.S., Colombo M., Craxì A., Donato F., Ferrari C., Gaeta G.B. (2008). Statements from the Taormina expert meeting on occult hepatitis B virus infection. J. Hepatol..

[3] Raimondo G., Locarnini S., Pollicino T., Levrero M., Zoulim F., Lok A.S. (2019). Taormina Workshop on Occult HBV Infection Faculty Members. Update of the statements on biology and clinical impact of occult hepatitis B virus infection. J. Hepatol..

[4] Mussa A., Ismail N.H., Huda Syed N., Talib M., Siddig A., Hassan R., Mohamud R., Haszroel N., Hashim H.N. (2024). Immune response to occult hepatitis B virus infection among hemodialysis patients: A review. Microbe.

[5] Bucio-Ortiz L., Enriquez-Navarro K., Maldonado-Rodríguez A., Torres-Flores J.M., Cevallos A.M., Salcedo M., Lira R. (2024). Occult Hepatitis B Virus Infection in Hepatic Diseases and Its Significance for the WHO’s Elimination Plan of Viral Hepatitis. Pathogens.

[6] Sun H., Mei L., Song S., Su Q., Yan Y., Ji H., Ma J., Chang L., Wang L. (2025). Potential oncogenic role of occult hepatitis B virus pre-S mutations: Activation of Akt/mTOR/Cyclin D1 signaling drives cell cycle dysregulation and proliferation in hepatocellular carcinogenesis. Genes Dis..

[7] Wati M.E., Yusra (2026). Occult hepatitis B infection. Hepatol. Forum.

[8] Vutien P., Nguyen M.H. (2025). HBV Reactivation in Immunosuppressed Patients: Screening, Prevention, and Management Including Solid Organ Transplant Recipients. Viruses.

[9] Bernieh B. (2015). Viral hepatitis in hemodialysis: An update. J. Transl. Intern. Med..

[10] Kvashnina D.V., Kovalishena O.V., Saperkin N.V. (2019). Risk factors of hepatitis B in patients on hemodialysis: A systematic review. Fundam. Clin. Med..

[11] Pranto A.H., Suez E., Uddin M.E., Tonmoy H.S., Meem M.M.R.M., Montasir F., Nourin F., Ahmed F., Abir S.N., Islam D.Z. (2026). Global epidemiology of hepatitis B virus infection in dialysis patients: A systematic review and meta-analysis of prevalence, immunity, risk factors, and beyond. Int. Urol. Nephrol..

[12] Light C., Heslop K., Kulkarni H. (2024). Comparison of Factors Affecting the Immune Response to Hepatitis B Vaccination in Patients with Stage 5 Chronic Kidney Disease-haemodialysis and Predialysis. Open Urol. Nephrol. J..

[13] Lürken K., Meinecke A., Manthey L.A., Cossmann A., Stankov M.V., Klawonn F., Zychlinsky Scharff A., Steffens S., Dopfer-Jablonka A., Müller F. (2025). Impaired Hepatitis B and COVID-19 vaccination responses show strong concordance in hemodialysis patients with end stage renal disease. Eur. J. Med. Res..

[14] Nacasch N., Cohen-Hagai K., Tayar N., Levian A., Rashid G., Benchetrit S., Neumark E., Wand O., Hod T., Rosman Y. (2022). Humoral Response to Hepatitis B and COVID-19 Vaccine among Maintenance Hemodialysis Patients. Vaccines.

[15] Lu K., Chiu K.Y. (2023). Lifetime Risk of Hepatorenal Events Assessed in Longitudinal Cohort of Renal Transplant Recipients with HBV Infection. Transpl. Proc..

[16] Fontenele A.M., Filho N.S., Ferreira A.S. (2013). Occult hepatitis B in patients on hemodialysis: A review. Ann. Hepatol..

[17] Tang Y., Liu X., Lu X., He Q., Li G., Zou Y. (2020). Occult Hepatitis B Virus Infection in Maintenance Hemodialysis Patients: Prevalence and Mutations in “a” Determinant. Int. J. Med. Sci..

[18] Ji D.Z., Pang X.Y., Shen D.T., Liu S.N., Goyal H., Xu H.G. (2022). Global prevalence of occult hepatitis B: A systematic review and meta-analysis. J. Viral Hepat..

[19] Nahid-Samiei R., Shokri S., Mahmoudvand S., Makvandi M., Shahbazian H., Shayanpur S., Nowrozi S., Hoseini Nejad K. (2018). Occult hepatitis B virus infection among hemodialysis patients. J. Biol. Res..

[20] Eltom K., Albeely A., El Hussein A.R.M., Elkhidir I.M., Enan K. (2020). Occult hepatitis B virus infection in Sudan: A systematic review and meta-analysis. JGH Open.

[21] Msomi N., Ndlovu K., Giandhari J., Wilkinson E., Parboosing R., Zungu S., Mlisana K. (2019). High rate of occult hepatitis B virus infection in hemodialysis units of KwaZulu-Natal, South Africa. J. Med. Virol..

[22] Ostankova Y.V., Serikova E.N., Semenov A.V., Bancevic M.D., Filipovic-Vignjevic S.B., Zueva E.B., Vasil’eva G.V., Zarya Y.V., Saitgalina M.A., Ivanova A.R. (2021). Profile of Hepatitis B Virus Mutations Associated with HBsAg-Negative Disease in Patients of Hemodialysis Centers. Probl. Part. Danger. Infect..

[23] Fabrizi F., Cerutti R., Alfieri C.M., Ridruejo E. (2021). An Update on Hepatocellular Carcinoma in Chronic Kidney Disease. Cancers.

[24] Martín Hidalgo-Barquero M.V., Ruiz-Calero Cendrero R.M., Cancho Castellano B., Cid Parra M.C., Galán González J. (2020). Chemotherapy and occult reactivation of HBV in hemodialysis patients. Nefrol. Engl. Ed. Engl. Span..

[25] Fu M.X., Elsharkawy A., Healy B., Jackson C., Bradshaw D., Watkins E., Ushiro-Lumb I., Griffiths J., Neuberger J., Maguire K. (2025). Occult hepatitis B virus infection: Risk for a blood supply, but how about individuals’ health?. eClinicalMedicine.

[26] Bourdin J., Sellier P., Salmona M., Lascoux-Combe C., Delaugerre C., Maylin S. (2024). Does the ultrasensitive HBsAg Next assay enhance Hepatitis B diagnosis? An evaluation of analytical performances. J. Clin. Virol..

[27] Daşdemir F.O., Dinç H.Ö., Sirekbasan S., Hamzeli N., Alaçam S., Karabulut N., Türk S., Dereli N., Akçin R., Karahan K.D. (2026). Okült HBV Enfeksiyonunda ve Düşük Düzey HBsAg Pozitifliğinde Yeni Nesil HBsAg Testinin Konvansiyonel Yöntemle Karşılaştırmalı Değerlendirilmesi [Comparative Evaluation of a New-Generation HBsAg Assay versus a Conventional Method in Occult HBV Infection and Low-level HBsAg Positivity]. Mikrobiyol. Bul..

[28] Coffin C.S., Vienneau J., Cooper C.L., Doucette K.E., Elkashab M., Ko H.H., Ramji A., Tam E., Fung S.K., Congly S.E. (2025). Ultrasensitive detection of HBsAg and HBV genome analysis in a prospective cohort with chronic hepatitis B after HBV surface antigen loss. Hepatol. Commun..

[29] Makvandi M. (2016). Update on occult hepatitis B virus infection. World J. Gastroenterol..

[30] Zhang Z., Zhang L., Dai Y., Zhang Y., Li J., Li X. (2016). Occult hepatitis B virus infection: Influence of S protein variants. Virol. J..

[31] Martin C.M., Welge J.A., Rouster S.D., Shata M.T., Sherman K.E., Blackard J.T. (2012). Mutations associated with occult hepatitis B virus infection result in decreased surface antigen expression in vitro. J. Viral Hepat..

[32] Mbencho M.N., Hafza N., Cao L.C., Mingo V.N., Achidi E.A., Ghogomu S.M., Velavan T.P. (2024). Incidence of Occult Hepatitis B Infection (OBI) and hepatitis B genotype characterization among blood donors in Cameroon. PLoS ONE.

[33] Jiang X., Chang L., Yan Y., Ji H., Sun H., Xiao Y., Song S., Feng K., Nuermaimaiti A., Wang L. (2022). Role of S protein transmembrane domain mutations in the development of occult hepatitis B virus infection. Emerg. Microbes Infect..

[34] Anderson M., Choga W.T., Moyo S., Bell T.G., Mbangiwa T., Phinius B.B., Bhebhe L., Sebunya T.K., Makhema J., Marlink R. (2018). In Silico Analysis of Hepatitis B Virus Occult Associated Mutations in Botswana Using a Novel Algorithm. Genes.

[35] Eslami N., Poortahmasebi V., Sadeghi J., Ghotaslou R., Niknafs B., Bannazadeh Baghi H., Ahangar Oskouee M. (2022). Occult Hepatitis B Infection among Hemodialysis in Tabriz, Northwest of Iran: Prevalence and Mutations within the S Region. Can. J. Infect. Dis. Med. Microbiol..

[36] Zou Y., Chen S., Cui Y., Zou Y. (2024). M133S mutation possibly involve in the ER stress and mitophagy pathway in maintenance hemodialysis patients with occult hepatitis B infection. Sci. Rep..

[37] Rastegarvand N., Makvandi M., Samarbafzadeh A., Rasti M., Neisi N., Pouremamali A., Teimoori A., Shabani A. (2015). Molecular Characterization of Pre-Core/Core and S Region of Hepatitis B Virus in Hemodialysis Patients with Occult Hepatitis B Infection. Jundishapur J. Microbiol..

[38] Mo Y., Jin F., Li D., Zou W., Zhong J., Tong Z., Wang W., Qian F. (2024). Prevalence and molecular characteristics of occult hepatitis B virus infection among blood donors in Huzhou City, eastern China. Gene.

[39] Schlabe S., van Bremen K., Aldabbagh S., Glebe D., Bremer C.M., Marsen T., Mellin W., Cristanziano V.D., Eis-Hübinger A.M., Spengler U. (2018). Hepatitis B virus subgenotype F3 reactivation with vaccine escape mutations: A case report and review of the literature. World J. Hepatol..

[40] Wang M., Xu R., Huang J., Liao Q., Tang X., Shan Z., Zhong H., Rong X., Fu Y. (2022). Molecular characteristics of the full-length genome of occult hepatitis B virus from blood donors in China. Sci. Rep..

[41] Boumbaly S., Balde T.L., Semenov A.V., Ostankova Y.V., Serikova E.N., Naidenova E.V., Valutite D.E., Shchemelev A.N., Zueva E.B., Esaulenko E.V. (2022). Prevalence of viral hepatitis B markers among blood donors in the Republic of Guinea. Probl. Virol..

[42] Ostankova Y.V., Shchemelev A.N., Serikova E.N., Kusevitskaya M.B., Sannikov M.V., Gorskaya O.A., Basina V.V., Shirshova N.Y., Mashkov I.A., Zueva E.B. (2025). Prevalence and Molecular Characterization of Chronic and Occult Hepatitis B Virus Infection Among Pregnant Women in St. Petersburg, Russia. Int. J. Mol. Sci..

[43] Thu H.H.K., Ostankova Y.V., Shchemelev A.N., Serikova E.N., Davydenko V.S., Ton T., Lien T.T.X., Ramsay E.S., Totolian A.A. (2026). Unveiling the Hidden Reservoir: High Prevalence of Occult Hepatitis B and Associated Surface Gene Mutations in a Healthy Vietnamese Adult Cohort. Microorganisms.

[44] Samadi E., Mirshahabi H., Motamed N., Sadeghi H. (2020). Prevalence of Occult Hepatitis B Virus Infection in Hemodialysis Patients Using Nested PCR. Rep. Biochem. Mol. Biol..

[45] Wang C., Xue R., Wang X., Xiao L., Xian J. (2023). High-sensitivity HBV DNA test for the diagnosis of occult HBV infection: Commonly used but not reliable. Front. Cell Infect. Microbiol..

[46] Serikova E.N., Ostankova Y.V., Shchemelev A.N., Pechnikova N.A., Ramsay E.S., Totolian A.A. (2026). Genetic Diversity, Occult Hepatitis B, and Mutational Signatures in Migrants from Regions with Varying HBV Endemicity: Importation of Diverse Viral Variants into St. Petersburg, Russia. Int. J. Mol. Sci..

[47] Ramachandran S., Zhai X., Thai H., Campo D.S., Xia G., Ganova-Raeva L.M., Drobeniuc J., Khudyakov Y.E. (2011). Evaluation of intra-host variants of the entire hepatitis B virus genome. PLoS ONE.

[48] Chook J.B., Teo W.L., Ngeow Y.F., Tee K.K., Ng K.P., Mohamed R. (2015). Universal Primers for Detection and Sequencing of Hepatitis B Virus Genomes across Genotypes A to G. J. Clin. Microbiol..

[49] Wang X., Xu L., Chen Y., Liu A., Wang L., Xu P., Liu Y., Li L., Meng F. (2017). Integrating nested PCR with high-throughput sequencing to characterize mutations of HBV genome in low viral load samples. Medicine.

[50] Hebeler-Barbosa F., Wolf I.R., Valente G.T., Mello F.C.D.A., Lampe E., Pardini M.I.M.C., Grotto R.M.T. (2020). A New Method for Next-Generation Sequencing of the Full Hepatitis B Virus Genome from A Clinical Specimen: Impact for Virus Genotyping. Microorganisms.

[51] Boumbaly S., Serikova E.N., Balde T.A.L., Ostankova Y.V., Schemelev A.N., Valutite D.E., Zueva E.B., Semenov A.V., Totolian Areg A. (2021). Amino acid substitutions in core and HBsAg regions of hepatitis B virus in patients with monoinfection and HBV/HIV-coinfection in the Republic of Guinea. HIV Infect. Immunosuppr. Disord..

[52] Okonechnikov K., Golosova O., Fursov M., The UGENE Team (2012). Unipro UGENE: A unified bioinformatics toolkit. Bioinformatics.

[53] Tamura K., Stecher G., Kumar S. (2021). MEGA11: Molecular Evolutionary Genetics Analysis Version 11. Mol. Biol. Evol..

[54] HIV-GRADE, HBV. https://www.hiv-grade.de/cms/grade/results/hbv.

[55] Genafor Geno2pheno HBV. https://hbv.geno2pheno.org/.

[56] Park S., Park E.S., Koo J.E., Park Y.K., Lee A.R., Dezhbord M., Cho E.S., Ahn S.H., Kim D.H., Lee J.H. (2020). Entecavir-resistant hepatitis B virus decreases surface antigenicity: A full genome and functional characterization. Liver Int..

[57] Sowole L., Labbett W., Patel M., O’Riordan A., Cross J., Davenport A., Haque T. (2015). The prevalence of occult hepatitis B virus (HBV) infection in a large multi-ethnic haemodialysis cohort. BMC Nephrol..

[58] Kalantari H., Ferdowsi F., Yaran M. (2016). Prevalence of occult hepatitis B virus infection in hemodialysis patients in Isfahan, Iran. Adv. BioMed. Res..

[59] Farshadpour F., Taherkhani R., Shahabi A. (2023). High Prevalence of Occult Hepatitis B Virus Infection among Iranian Hemodialysis Patients. Am. J. Trop. Med. Hyg..

[60] Musa M.D., Ateya H.K. (2020). Prevalence of overt and occult hepatitis B virus infections among 135 haemodialysis patients attending a haemodialysis centre at Al-Nasiriyah city, Iraq. Iran. J. Microbiol..

[61] Bokharaei-Salim F., Keyvani H., Monavari S.H., Esghaei M., Fakhim S., Ataei Pirkooh A., Behnava B. (2014). Distribution of hepatitis B virus genotypes in azerbaijani patients with chronic hepatitis B infection. Hepat. Mon..

[62] Masoodi I., Singh C., Wani I.A., Wani M.M., Ahmed T.I., Sheikh R.Y. (2019). Sero Conversion of Viral Hepatitis among End Stage Renal Disease Patients on Hemodialysis in Kashmir: Results of a Prospective Study. Open Access Maced. J. Med. Sci..

[63] Fujikura T., Isobe S., Oikawa S., Ishigaki S., Katahashi N., Iwakura T., Ohashi N., Kato A., Yasuda H. (2025). Comparison of seroconversion rates after hepatitis B vaccination in patients with advanced chronic kidney disease and those receiving maintenance hemodialysis. Clin. Exp. Nephrol..

[64] Islam M.N., Hafizur Rahman G.M., Faisal A.R., Juthy S., Amin N., Sultana S., Jannat G., Islam Z. (2025). WCN25-2742 Immune response to hepatitis B vaccination in CKD patients and factors associated with non-response. Kidney Int. Rep..

[65] Yan B., Lv J., Feng Y., Liu J., Ji F., Xu A., Zhang L. (2017). Temporal trend of hepatitis B surface mutations in the post-immunization period: 9 years of surveillance (2005–2013) in eastern China. Sci. Rep..

[66] Colagrossi L., Hermans L.E., Salpini R., Di Carlo D., Pas S.D., Alvarez M., Ben-Ari Z., Boland G., Bruzzone B., Coppola N. (2018). Immune-escape mutations and stop-codons in HBsAg develop in a large proportion of patients with chronic HBV infection exposed to anti-HBV drugs in Europe. BMC Infect. Dis..

[67] Hossain M.G., Ueda K. (2019). A meta-analysis on genetic variability of RT/HBsAg overlapping region of hepatitis B virus (HBV) isolates of Bangladesh. Infect. Agent Cancer.

[68] Logoida M., Kristian P., Schreiberova A., Lenártová P.D., Bednárová V., Hatalová E., Hockicková I., Dražilová S., Jarčuška P., Janičko M. (2021). Comparison of Two Diagnostic Methods for the Detection of Hepatitis B Virus Genotypes in the Slovak Republic. Pathogens.

[69] Motta J.S., Mello F.C., Lago B.V., Perez R.M., Gomes S.A., Figueiredo F.F. (2010). Occult hepatitis B virus infection and lamivudine-resistant mutations in isolates from renal patients undergoing hemodialysis. J. Gastroenterol. Hepatol..

