# Supplementary material for: Overt and Occult Hepatitis B in Hemodialysis Patients: Insights from High-Sensitivity Testing and Genetic Analysis
Source: Diagnostics (Basel). 2026 Oct 30;16(21):3588. doi: 10.3390/diagnostics16213588 (PMC13762082; doi:10.3390/diagnostics16213588)
Supplement: Supplementary file 1 [file diagnostics-16-03588-s001.zip › diagnostics-4588956-supplementary.pdf]

Supplementary Table S1. Thermal cycling conditions for the first round of nested PCR.

Block	Step	Temperature (°C)	Time	Number of cycles
1	Initial denaturation	95	5 min	1
2	Denaturation	95	20 sec	20
	Annealing	52	30 sec	
	Extension	72	3 min 50 sec	
3	Final extension	72	7 min	1
4	Denaturation	95	20 sec	20
	Annealing	52	30 sec	
	Extension	72	3 min 50 sec	
5	Final extension	72	7 min	1
6	Denaturation	95	20 sec	10
	Annealing	50	30 sec	
	Extension	72	3 min 50 sec	
7	Final extension	72	7 min	1
8	Hold	4	∞	–

Supplementary Table S2. Thermal cycling conditions for the second round of nested PCR.

Block	Step	Temperature (°C)	Time	Fluorescence reading	Number of cycles
1	Initial denaturation	95	5 min	–	1
2	Denaturation	95	10 s	–	7
	Annealing	50	20 s	–	
	Extension	72	40 s	–	
3	Final extension	72	5 min	–	1
4	Denaturation	95	10 s	–	45
	Annealing	58	20 s	FAM, ROX, CY5	
	Extension	72	40 s	–	
5	Hold	4	∞	–	–
